# Mitochondrial anchor protein Num11 is key to pathogenicity of *Candida albicans* by affecting mitochondrial function and cell wall masking

**DOI:** 10.1080/21505594.2025.2519149

**Published:** 2025-06-18

**Authors:** Guangyuan Yang, Xiaojia Niu, Tian Zhuang, Xiaoxiao Zhu, Qianwen Xu, Hongchen Wang, Jing Shao, Changzhong Wang, Yue Yang, Tianming Wang, Wenfan Wei, Daqiang Wu

**Affiliations:** a Department of Pathogenic Biology and Immunology, College of Integrated Chinese and Western Medicine, Anhui University of Chinese Medicine, Hefei, China; b Key laboratory of Xin’an Medicine, Ministry of Education, Research Institute of Integrated Traditional Chinese and Western Medicine, Anhui Academy of Chinese Medicine, Hefei, China

**Keywords:** *Candida albicans*, anchor protein Num11, mitochondria, β-glucan surface exposed, Cdc42-Cek1 pathway, pathogenicity

## Abstract

The mitochondrial anchoring protein Num1 directly affects mitochondrial redox function, cell division, and growth in unicellular fungi. However, the functional characterization of Num11, its *Candida albicans* homolog, remains elusive. Our investigation revealed that Num11 deletion in *C. albicans* caused profound cellular defects: (1) Disrupted cell cycle progression and mitochondrial dysfunction manifesting as mitochondrial morphological aggregation, ATP depletion, membrane potential collapse, and ROS overproduction; (2) Hypersensitivity to cell wall-perturbing agents accompanied by thicker cell walls and increased surface exposure of β-glucan/chitin; (3) Enhanced macrophage phagocytosis and proinflammatory cytokine release. These cellular alterations translated to significantly attenuated virulence in both *Galleria mellonella* and systematic mice infection models. Mechanistically, transcriptome profiling and protein interaction analyses demonstrated Num11 deficiency hyperactivates the Cdc42-Cek1 MAPK cascade (phospho-Cek1 increased), driving cell wall remodeling. Our findings establish Num11’s dual closely connected regulatory roles in *C. albicans* pathogenesis: as a mitochondrial scaffold maintaining bioenergetic homeostasis to attenuate growth and as a negative regulator of the Cdc42-Cek1 axis controlling cell wall architecture through affection on mitochondria. These coordinated actions collectively underscore Num11’s critical role in mediating host–pathogen interactions during invasive candidiasis.

## GRAPHICAL ABSTRACT

**Figure:**
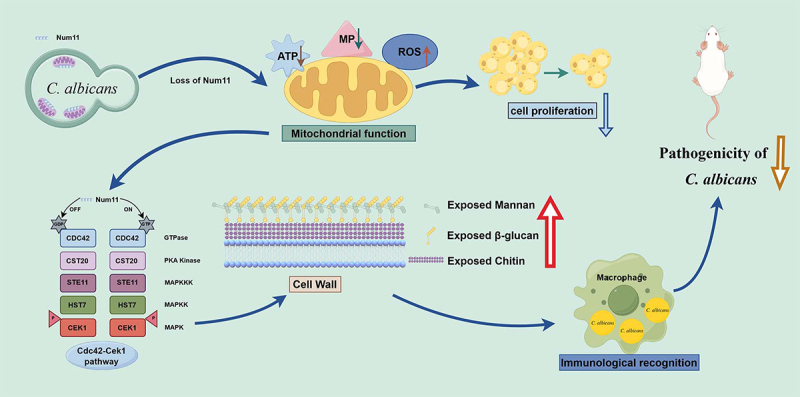

## Introduction

*Candida albicans* serves as a model organism and is a widespread opportunistic pathogen linked to various human diseases, commonly colonizing the mucosal surfaces of the gastrointestinal tract, reproductive tract, oral cavity, and skin in healthy individuals [1]. In individuals with a strong immune system, *C. albicans* typically remains harmless, existing in balance with the host’s microbiota [2]. However, changes in the host environment, such as shifts in pH, nutritional composition, or immune response caused by stress, other infections, metabolic disorders, or immunosuppressive treatments, can lead to excessive growth and filamentation of *C. albicans* [3]. This overgrowth can cause infections ranging from minor to severe, including potentially fatal bloodstream infections with mortality rates approaching 40% in certain cases [4]. The overuse of antifungal medications has resulted in increasing drug resistance among *C. albicans*, posing a significant challenge for current antifungal therapies [5,6]. Therefore, it is crucial to investigate the biological characteristics of *C. albicans* and to identify new antifungal therapeutic target genes involved in their pathogenicity and drug resistance.

All eukaryotic species contain a special organelle in their cells, namely mitochondria, which serve as the primary regulator of metabolism [7]. Mitochondria play crucial roles across a wide spectrum of eukaryotic cell physiology. In pathogenic fungi, this vital metabolic organelle orchestrates various functions linked to disease, including the pathogen’s fitness, developmental and morphogenetic transitions, and susceptibility to antifungal drugs [7]. The main function of mitochondria is to provide energy through the respiratory chain [8,9]. In pathogenic fungi including *C. albicans*, *Cryptococcus neoformans*, and *Aspergillus fumigatus*, inhibiting the respiratory chain can affect the morphological transition from yeast to hyphae, cell wall biogenesis, drug resistance, and virulence [10–17]. Recently, our group found that Oct1, a protease found in mitochondria, manages mitochondrial stability and affects disease-causing potential by altering hyphal growth and biofilm formation in *C. albicans* [18]. The respiratory chain has been proposed as an attractive target for developing antifungal agents to alleviate fungal infections and prevent the evolution of drug resistance [19]. Thus, a deeper understanding of mitochondrial biology in invasive fungal pathogens, especially *C. albicans*, is needed.

The fungal cell wall is mainly made up of β-glucans, chitin and mannoproteins, and its composition varies among different species [20]. This complex polysaccharide matrix surrounds the fungal cytoplasm. It protects the cell from changes in external osmotic pressure, pH, and other physical and chemical conditions. The fungal cell wall is a vital structure that directly interacts with host cells, playing a key role in the pathogenicity of invasive fungi [21,22]. The cell wall constitutes a highly dynamic structure, and its disruption exerts a profound impact on the survival of fungal cells, culminating in plasma membrane rupture and subsequent cell lysis [23]. The activity to mask the immunogenic cell wall especially β-glucan which also called pathogen-associated molecular pattern from host immune recognition significantly enhances fungal virulence [24]. The processes of cell wall biosynthesis and remodeling are regulated by intricate signaling pathways, notably including several mitogen-activated protein kinase (MAPK) cascades and their upstream Rho-type GTPases [25]. Within these MAPK pathways, the Ste11-Hst7-Cek1 components constitute the Cek1-MAPK cascade, which has been documented to regulate β-glucan surface exposed in *C. albicans* [26]. Todd and colleagues documented that the excessive activation of the Cek1 MAP kinase cascade aids in exposing the cell wall, consequently boosting the immune response provoked by the strain [27]. Elevating Cek1’s activity in a mouse model of infection led to decreased fungal virulence, which correlated with heightened cytokine production from macrophages [27]. Furthermore, Lrg1, a putative GTPase-activating protein (GAP), inhibits Cek1’s activity by suppressing the function of the GTPase Cdc42 and its subsequent MAPKKK, Ste11 [28]. Currently, there is a lack of studies examining how mitochondrial-associated proteins affect fungal cell wall components, particularly regarding the Cdc42-Cek1 signaling pathway.

Fungal cells, similar to mammalian cells, demonstrate intricate interactions between their organelles and the plasma membrane through specialized contact sites [29]. A specific example is ERMES, a tether connecting the endoplasmic reticulum and mitochondria, which plays a role in evading the immune system by modulating inflammasome responses to hyphal signals in *C. albicans* [30]. Notably, research has identified the mitochondria-endoplasmic reticulum-cortex anchor (MECA) in *S. cerevisiae* as a critical structure that links mitochondria with the endoplasmic reticulum and plasma membrane [31]. MECA functions to sustain mitochondria in regions with elevated demand for mitochondrial activity, thereby influencing the quantity and quality of mitochondria inherited by daughter cells [31]. Consequently, the spatial and temporal positioning of mitochondria, facilitated by anchoring mechanisms, is intricately linked to cellular functions and physiology [32]. Recent studies have identified that the MECA complex comprises at least two proteins (Num1 and Mdm36) and three organelles (mitochondria, endoplasmic reticulum, and plasma membrane) [33]. The principal element of MECA’s architecture is the membrane-associated anchoring protein Num1, which is integral to the division and positioning of mitochondria and nuclei [34]. The deficiency of Num1 results in aberrant mitochondrial division, leading to an irregular, network-like distribution within the cells [35]. In *S. cerevisiae*, Num1 is a 313 kD protein characterized by an N-terminal coiled-coil (CC) domain that directly interacts with the mitochondrial membrane, while the C-terminal pleckstrin homology (PH) domain facilitates the targeting of mitochondria to the plasma membrane [33,36]. Recent study has demonstrated that the absence of Num1 protein can result in defects in dynein anchoring, subsequently inhibiting mitochondrial respiratory function and cell division [37]. Additionally, a homologous functional protein, NuMA, has been identified in mammals [38], where it anchors the Dynein protein to exert a pulling force during spindle separation in mitosis, but cannot anchor to mitochondria. Thus, the mitochondria anchor protein Num1 is conserved and worth to investigate in other pathogenic fungi.

In the *Candida* Genome Database, we identified the highly homologous genes C4_06130W_A/B with *Num1*, designated as *NUM11*, with a total open reading frame (ORF) length of 11,481 base pairs, encoding a protein (Num11) consisting of 3,826 amino acids. Through protein structural analysis, we identified that Num11 comprises CC and PH domains which are analogous to those present in the Num1 protein in *S. cerevisiae*. Thus, the predicted biological functions of Num11 are similar to those of its homologs, Num1. However, unlike Num1 in *S. cerevisiae*, the subcellular localization and especially functional role in pathogenicity of Num11 in *C. albicans* remain inadequately characterized. Therefore, we can reasonably hypothesize that Num11 in *C. albicans* possesses similar biological functions to Num1 in *S. cerevisiae*. Additionally, it is worth to explore its potential impact on virulence-related masking of cell wall components in *C. albicans*.

To elucidate Num11’s biological functions, we constructed isogenic knockout (*num11*Δ/Δ) and complemented strains in *C. albicans*. Systematic characterization revealed three key phenotypes: (1) mitochondrial dysfunction evidenced by mitochondria aggregation, ATP depletion, and ROS accumulation; (2) compromised virulence determinants including reduced biofilm biomass; (3) cell wall remodeling featuring thicker walls (TEM analysis), increased surface β-glucan/chitin exposure (flow cytometry), and consequent higher macrophage phagocytosis rates. These *in vitro* findings correlated with significantly attenuated pathogenicity in infection models. Mechanistically, RNA-seq profiling identified hyperactivation of the Cdc42-Cek1 MAPK cascade in *num11*Δ/Δ, evidenced by increased Cek1 phosphorylation. This signaling alteration may result in increased surface exposed of polysaccharides of cell wall, such as β-glucan, in *C. albicans*. Our work establishes Num11 as a pleiotropic regulator coordinating mitochondrial homoeostasis with cell wall architecture via Cek1-mediated signaling, revealing novel therapeutic targets for antifungal development through pharmacological modulation of this mitochondria-to-cell wall communication axis.

## Results

### NUM11 gene knockout affects growth of *C. albicans*

**Figure 1.**
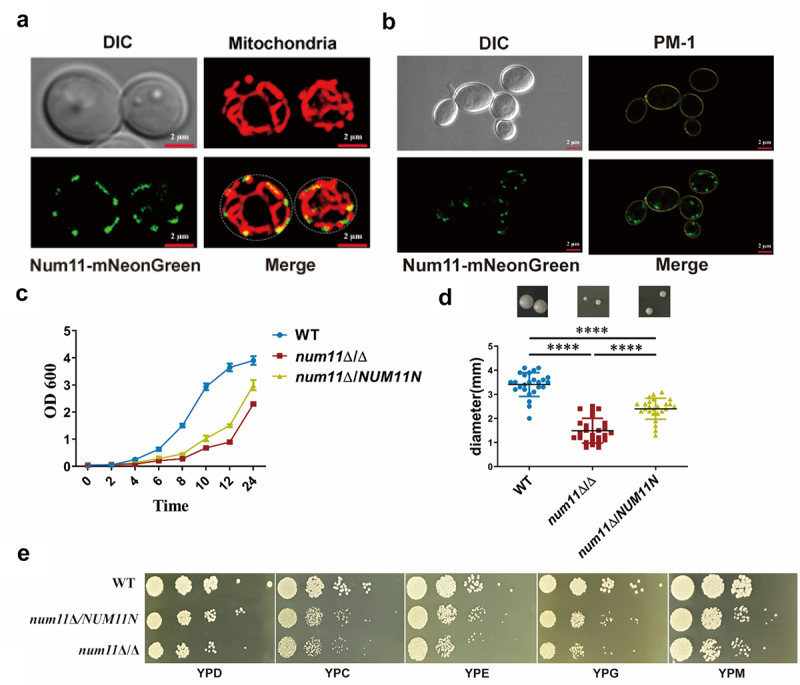
The subcellular localization of mitochondrial anchor protein Num11 and its impact on the growth of strains after gene deletion.

In *S. cerevisiae*, Num1 is localized at the cell membrane and demonstrates co-localization with mitochondria [33]. By constructing a Num11-mNeonGreen fusion expression strain (C-terminal tagged with an mNeonGreen tag) and using Mito-Tracker Red FM and plasma membrane-specific dye PM-1 in combination, we systematically analyzed the subcellular localization characteristics of the Num11 protein in *C. albicans*. Confocal microscopic analysis revealed (Figure 1a) that the fluorescence distribution of C-terminal of Num11 exhibited a colocalization with the mitochondria, confirming its biological characteristic as a mitochondrial anchoring protein. This is highly conserved in terms of localization pattern and function with the Num1 homolog protein in *S. cerevisiae* [33]. Notably, when dual-channel imaging was performed using the plasma membrane-specific dye PM-1 (Figure 1b), we found that no significant colocalization was detected between the Num11-mNeonGreen signal and the plasma membrane stain marker are not colocalized, but adjacent. The result suggests that C-terminal of Num11 is not located at plasma membrane, which is also consistent with Num1 in *S. cerevisiae* ^*33*^. These findings not only verify the evolutionary conservation of Num11 as a mitochondrial localization protein but also provide crucial cellular biological evidence for elucidating its regulatory role in pathogenesis through the mitochondrial-organelle interaction network.

(a) Determination of subcellular localization of Num11 with mitochondrial staining. Utilize fluorescent tags to label the anchoring protein homolog gene *NUM11* and employ Mito-Tracker to stain mitochondria for the purpose of analyzing their localization. Scale bar: 2 µm. (b) Determination of subcellular localization of Num11 with plasma membrane staining. Utilize fluorescent tags to label the anchoring protein homolog gene *NUM11* and employ PM-1 to stain plasma membrane for the purpose of analyzing their localization. Scale bar: 2 µm. (c) Growth curves of each strain. (d) Quantification of colony sizes of each strain. These strains were growth at 35°C on respiratory plate. The black line denotes the grand mean of at least three independent experiments. Inset images above the graph are representative images of quantified colonies for each strain. (e) Carbon source utilization of each strain in spot assay. The significance levels of *P* values were calculated by comparing with selected two groups as follows: ****P < 0.0001.

Due to the pivotal role of mitochondria in ATP production via cellular respiration, we knocked out the *NUM11* gene in *C. albicans*, and the detailed identification results of gene deletion are provided in Figure S1 and S2. We investigated cell growth on agar plates of *num11*Δ/Δ and *num11*Δ/*NUM11N*. Our analysis (Figure 1c) revealed that the *num11*Δ/Δ demonstrated significantly reduced growth rates and smaller single colonies compared to the WT parental strain. This growth impairment was observed across both fermentable carbon sources (Yeast Extract Peptone Dextrose Medium (YPD) and Yeast Extract Peptone Maltose medium (YPM)) and non-fermentable carbon sources (Yeast Extract Peptone Ethanol medium (YPE), Yeast Extract Peptone Citrate medium (YPC), and Yeast Extract Peptone Glycerol medium (YPG)). Importantly, the disparity in colony growth was more pronounced in non-fermentable carbon sources, indicating a more substantial growth defect under these conditions when fungal cell needs mitochondria to use non-fermentable carbon sources to produce energy. The complemented strain *num11Δ/NUM11N* exhibited a significantly growth recover on these agar plates (Figure 1e). To quantify the growth defect associated with the *num11*Δ/Δ strain, we measured the colony sizes of each strain at 35°C on non-fermentable carbon source plates. The results, presented in Figure 1d, indicate that the colony size of the *num11*Δ/Δ strain was significantly smaller compared to the parental WT strain. Thus, these findings suggest that the knockout of the *NUM11* gene substantially inhibits the growth rate and reproduction of *C. albicans*.

### Loss of Num11 causes dysfunction of mitochondria

**Figure 2.**
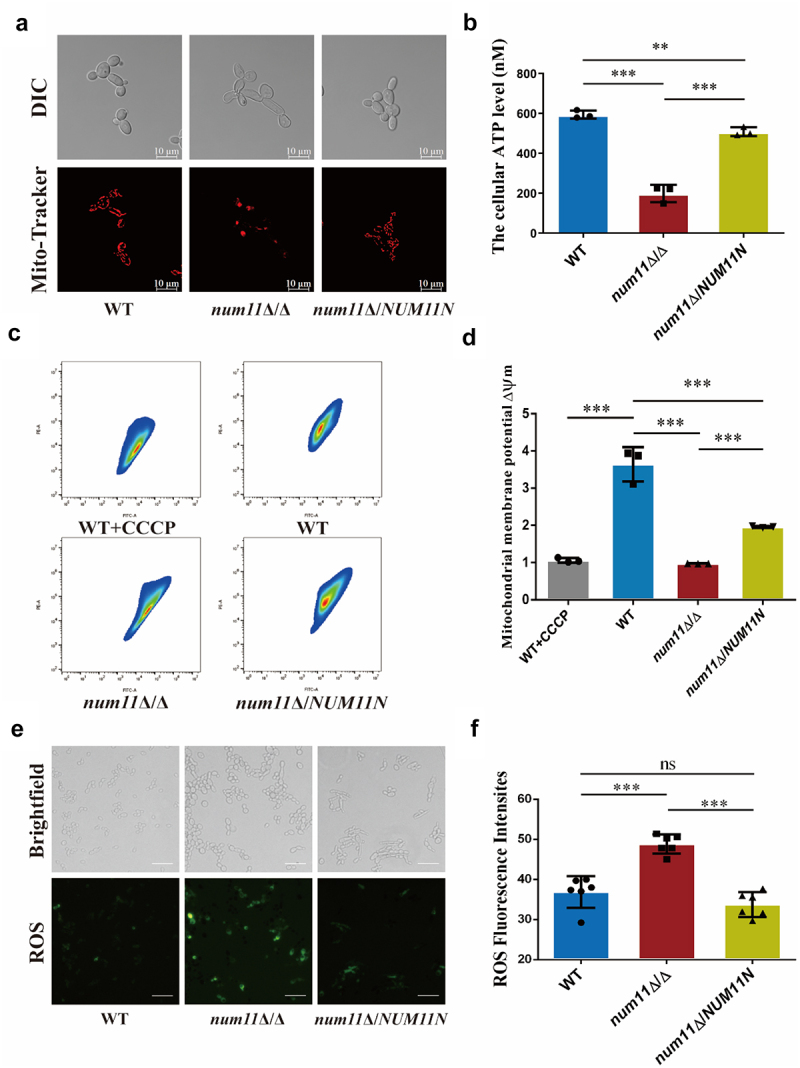
Loss of Num11 significantly inhibits *C. albicans* mitochondrial function.

Considering that the Num11 protein in *C. albicans* influences cell growth on non-fermentable carbon sources, we aimed to elucidate the effects of Num11 protein deficiency on mitochondrial function. Initial findings from Mito-Tracker staining (Figure 2a) indicated abnormalities in both mitochondrial morphology and localization in *num11*Δ/Δ strain. Morphologically, the WT strain exhibited 5–10 granular or small rod-shaped mitochondrial tubules that were discrete from one another. In contrast, the *num11*Δ/Δ strain displayed numerous tightly arranged tubules, the majority of which were interconnected, forming an irregular network structure. Additionally, fragmented tubules were also observed within the network in *num11*Δ/Δ cells. Regarding mitochondrial localization, the WT strain exhibited a regular and even distribution of mitochondria throughout the cell. In contrast, *num11*Δ/Δ cells displayed a disorganized mitochondrial distribution, characterized by a network structure frequently clustering on one side of the cell and fragmented structures dispersed irregularly. The complemented strain partially restored the mitochondrial morphology in *C. albicans* cells. Subsequently, we assessed the ATP content across various strains and observed a markedly lower ATP content in *num11*Δ/Δ cells compared to WT cells (Figure 2b). In contrast, the mitochondrial membrane potential in *num11*Δ/*NUM11N* cells exhibited a significant increase relative to *num11*Δ/Δ cells, although it did not completely return to baseline levels. Additionally, the results pertaining to mitochondrial membrane potential (Figure 2c,d) indicated variations in membrane potential levels among the different strains. Relative to the WT, the positive control group (WT+(Carbonyl cyanide 3-chlorophenylhydrazone CCCP)) demonstrated a significant reduction in mitochondrial membrane potential levels. Likewise, *num11*Δ/Δ cells exhibited a markedly decreased mitochondrial membrane potential compared to WT. Conversely, the mitochondrial membrane potential in *num11*Δ/*NUM11N* cells showed a significant increase relative to *num11*Δ/Δ cells. Subsequently, we assessed reactive oxygen species (ROS) levels in *C. albicans* cells (Figure 2e,f) and observed that *num11*Δ/Δ cells exhibited significantly elevated intracellular ROS levels compared to WT cells, with a high degree of statistical significance. In comparison to the *num11*Δ/Δ strain, the *num11*Δ/*NUM11*N cells exhibited a significant reduction in intracellular ROS levels. In conclusion, our findings suggest that the lack of the Num11 protein in *C. albicans* significantly changes mitochondrial morphology, reduces ATP production and membrane potential, and increases levels of ROS leading to dysfunction of mitochondria.

(a) Mitochondrial morphology and location of various strains. Scale bar: 10 µm. (b) The intracellular ATP level of each strain. (c) Fluorescent expression of mitochondrial membrane potential levels in various strains. Carbonyl cyanide 3-chlorophenylhydrazone (CCCP) was added according to the instruction of kit. (d) Average fluorescence intensity of mitochondrial membrane potential level for each strain. (e) Fluorescence images of ROS (DCFH-DA stained) for each strain. Scale bar: 40 µm. (f) Quantitative bar chart of ROS fluorescence. The significance levels of *P* values were calculated by comparing with the selected two groups as follows: **0.001<*P*<0.01 and ***0.0001<*P*<0.001. The “ns” represents no significant difference.

### Loss of Num11 affects biosynthesis and surface exposed of cell wall polysaccharide

**Figure 3.**
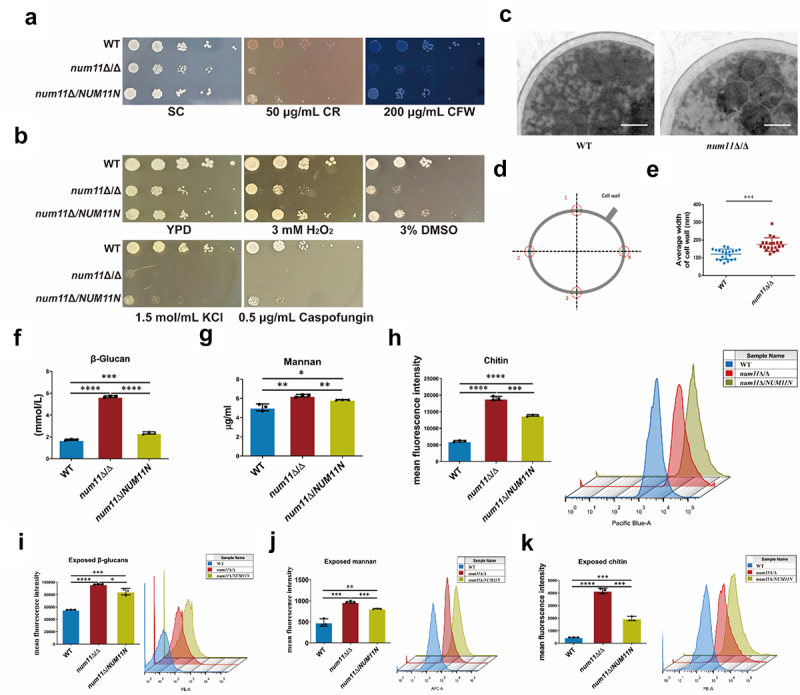
Loss of Num11 affects *C. albicans* cell wall structure.

The fungal cell wall is essential for the survival of fungal pathogens, as its chemical composition dictates immune recognition of *C. albicans* and serves as a target for widely used antifungal agent, echinocandins [24]. Considering that the Num11 protein functions as an anchor protein linking the mitochondria to cytomembrane and cell wall in *C. albicans*, we sought to examine whether Num11 affects the composition and formation of the cell wall in this species. Initially, a series of spot plate assays demonstrated that the *num11*Δ/Δ, exhibited increased sensitivity to cell wall stress agents, Congo Red (CR) and Calcofluor White (CFW), in comparison to the WT strain (Figure 3a). Additionally, these assays indicated that the *num11*Δ/Δ strain also showed heightened sensitivity to caspofungin (CAS) which is antifungal agent targeting cell wall biosynthesis, H_2_O_2_, DMSO, and KCl (Figure 3b). Based on these findings, we hypothesized that the absence of the Num11 could result in modifications to the properties and composition of the *C. albicans* cell wall.

(a) Cell susceptibility to cell wall-perturbing agents. (b) Detection of cell sensitivity to membrane-perturbing agents, drugs, and metal ions. The cells were incubated at 30°C for 2 days. A representative image from three independent experiments with identical results is shown. (c) Cell wall morphology of each strain. Scale bar: 500 nm. (d) Diagram of the cell wall thickness measurement method. (e) Average thickness of the cell wall for each strain. (*n* = 20) (f) Content of β-glucan extracted from each strain. (g) Detection of mannan in various strains. (h) Average fluorescent intensity (left) and representative flow cytometry profiles (right) of total amount of chitin assessed by CFW. (i) Average fluorescent intensity (left) and representative flow cytometry profiles (right) of CW β-glucan surface exposed Cells were incubated with primary anti-β-glucan antibody and PE-conjugated secondary antibody. (j) Average fluorescent intensity (left) and representative flow cytometry profiles (right) of CW mannan surface exposed assessed by Concanavalin A (Con A). (k) Average fluorescent intensity (left) and representative flow cytometry profiles (right) of CW chitin surface exposed assessed by wheat germ agglutinin. The significance levels of *P* values were calculated by comparing with selected two groups as follows: * 0.01 < *P* < 0.05, ** 0.001 < *P* < 0.01, *** 0.0001<*P* < 0.001 and ****P < 0.0001. The “ns” represents no significant difference.

Consequently, we employed transmission electron microscopy to examine whether the deletion of the *NUM11* gene induced morphological changes in the cell wall and to quantify the cell wall thickness (Figure 3c). The WT strain exhibits a compact cell membrane and a typical, relatively dense cell wall structure. Conversely, the *num11*Δ/Δ strain exhibits a loose cell membrane, increased cell wall thickness, and a less compact structure, markedly deviating from the normal cellular architecture. As illustrated in Figure 3e, the cell wall of the knockout strain *num11*Δ/Δ exhibited a statistically significant increase in thickness compared to the WT strain. Meanwhile, we also examined the expression levels of genes related to cell wall synthesis through quantitative real-time polymerase chain reaction, with the results presented in Figure S3. In the *num11*Δ/Δ strain, the expression levels of genes related to cell wall synthesis were all higher than those in the WT strain. In the *num11*Δ/*NUM11N* strain, except for the expression levels of the *PHR1* and *OCH1* genes, other seven cell wall synthesis related genes (especially *CHS2*, *ALG5*, *BGL2*, *GSC1*) were lower than those in the *num11*Δ/Δ strain, which is basically correlated with comparison of WT and *num11*Δ/Δ strains.

The principal polysaccharides in the cell wall of *C. albicans* include β-glucan, chitin, and mannoproteins. Consequently, we measured the content level of β-glucan, chitin, and mannoproteins in the WT, *num11*Δ/Δ, and *num11*Δ/*NUM11N* strains. As illustrated in Figure 3f-h, the *num11*Δ/Δ exhibited significantly elevated concentrations of β-glucan, chitin, and mannan in comparison to the WT strain. Furthermore, deleting the *NUM11* gene increased the surface exposed of β-glucan, chitin, and mannan in the cell wall of *C. albicans* (Figure 3i-k). In conclusion, these findings provide the first evidence that the absence of the mitochondrial anchor protein Num11 leads to increased cell wall thickness in *C. albicans*, along with higher polysaccharide content and significantly enhanced polysaccharide surface exposed.

### Loss of Num11 enhances macrophage recognition and phagocytosis of *C. albicans*

**Figure 4.**
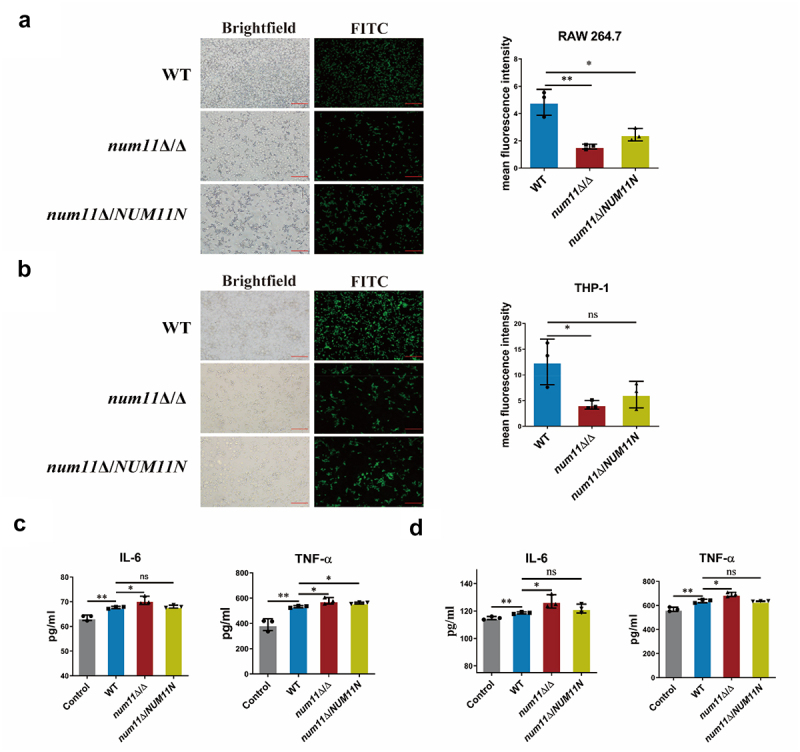
Loss of Num11 affects macrophage recognition and phagocytosis of *C. albicans* in vitro.

*C. albicans* can circumvent immune detection by host pattern recognition receptors through the masking of cell wall components, thereby facilitating immune evasion [39]. Due to that *C. albicans* cells with a deletion of the *NUM11* gene exhibit increased surface exposed of β-glucan, we hypothesize that these cells may be more readily recognized and phagocytosed by macrophages *in vivo*. To test this hypothesis, we co-incubated WT, *num11*Δ/Δ, and *num11*Δ/*NUM11N* strains with two macrophage cell lines (RAW264.7 and THP-1) with a macrophage to *C. albicans* ratio of 1:3 for a duration of 3 h. Phagocytosis was subsequently assessed through microscopic observation. The results (Figure 4a,b) indicated that the *num11*Δ/Δ exhibited increased susceptibility to recognition and phagocytosis by both RAW264.7 and THP-1 macrophages in comparison to the WT strain. Additionally, we quantified the secretion levels of the pro-inflammatory cytokines TNF-α and IL-6 in the supernatants of RAW264.7 and THP-1 macrophages. As illustrated in Figure 4c,d, the *num11*Δ/Δ elicited an enhanced secretion of pro-inflammatory cytokines TNF-α and IL-6 in RAW264.7 and THP-1 macrophages compared to the parental WT strain. These findings suggest that without the Num11 protein, *C. albicans* cells are easier for host macrophages to recognize and engulf and trigger an elevated host inflammatory response during infection.

(a) Representative fluorescent images (left) and intensity comparison (right) of RAW264.7 cell phagocytosing each strain. Scale bar: 20 µm. (b) Representative fluorescent images (left) and intensity comparison (right) of THP-1 cell phagocytosing each strain. Scale bar: 20 µm. (c) Comparison of secretion levels of the cytokines IL-6 and TNF-α of RAW 264.7 macrophages. (d) Comparison of secretion levels of the cytokines IL-6 and TNF-α of THP-1 macrophages. The significance levels of *P* values were calculated by comparing with the selected two groups as follows: * 0.01 < *P* < 0.05, ** 0.001 < *P* < 0.01 and *** 0.0001 < *P* < 0.001. The “ns” represents no significant difference.

### Deletion of the NUM11 gene affects virulence of *C. albicans* in vivo

**Figure 5.**
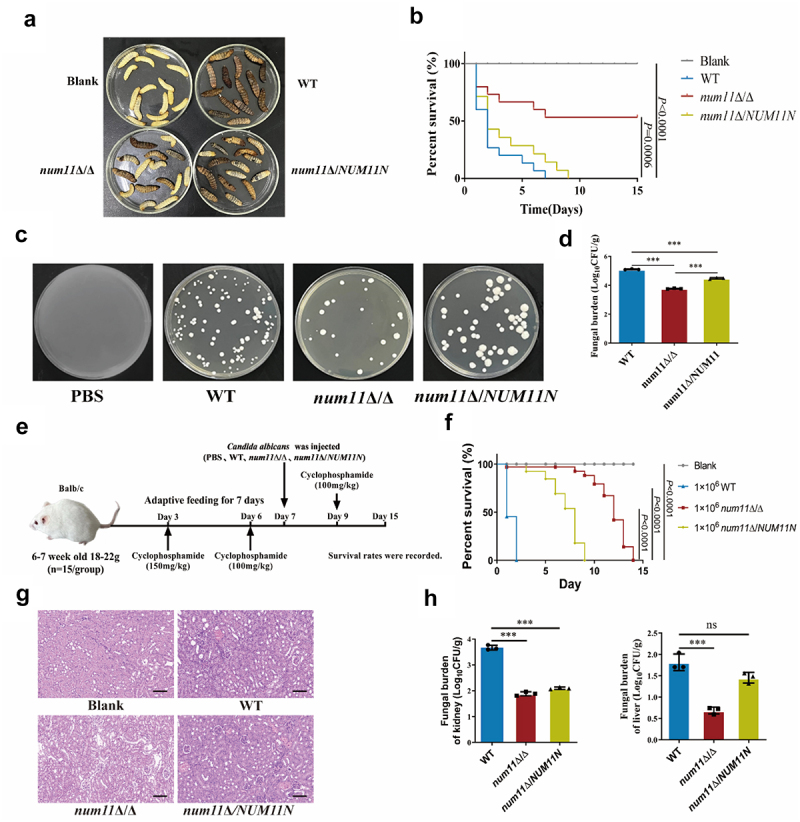
Loss of Num11 reduces the pathogenicity of *C. albicans* in vivo.

Our prior research demonstrated that the deletion of *NUM11* gene resulted in enhanced surface exposed of cell wall polysaccharides in *C. albicans*, which significantly indues the macrophage recognition and phagocytosis of *C. albicans*. Based on these observations, we speculated that the lack of Num11 would significantly reduce the pathogenicity of *C. albicans*. To evaluate this hypothesis, we initially performed infection assays utilizing *Galleria mellonella* larvae as an invertebrate model. The 14-day survival curve (Figure 5a,b) demonstrated that all larvae infected with the WT strain succumbed by day 7, whereas those infected with the *num11*Δ/Δ strain exhibited significantly higher survival rates, with approximately 50% surviving. Conversely, larvae infected with the *num11*Δ/*NUM11N* strain all perished by day 9. Fungal load analysis in the larvae (Figure 5c,d) revealed a significant reduction in fungal burden in *num11*Δ/Δ-infected larvae compared to those infected with the WT strain.

**Figure 6.**
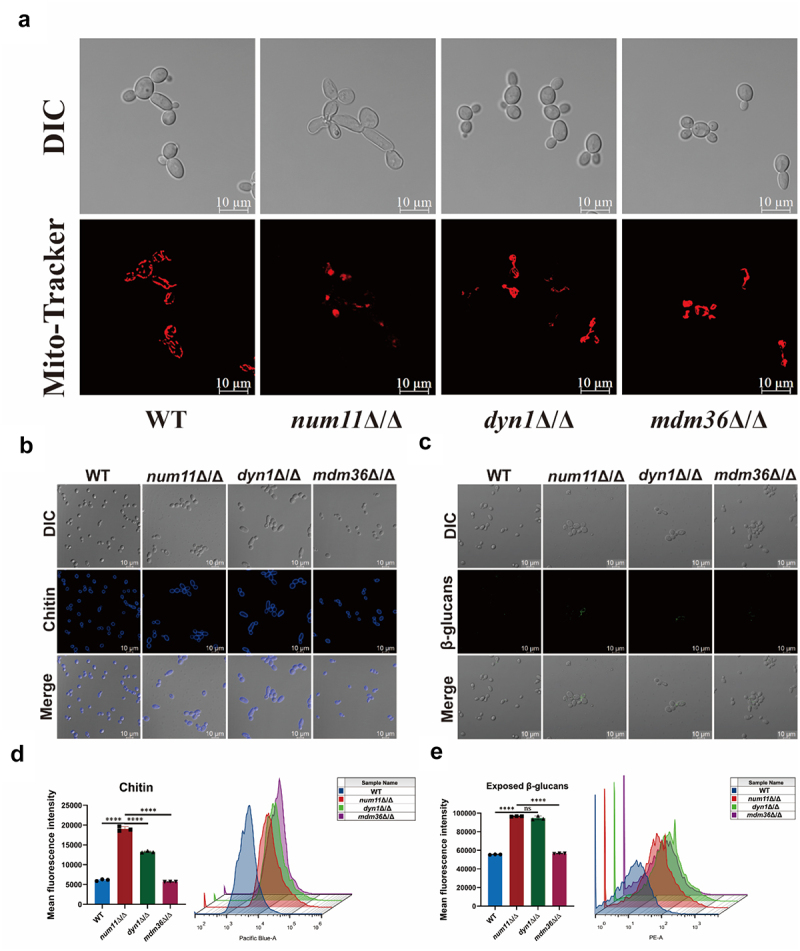
Comparing the impact of the loss of Num11, Mdm36, and Dyn1 on mitochondrial damage and surface exposed of β-glucan and chitin in *C. albicans*.

(a) Survival of *G. mellonella* for 15 days. (b) Survival curve of *G. mellonella*. (c-d) Fungal load in *G. mellonella*. (e) Schematic representation of the systemic infection model of *C. albicans* in mice. (f) Survival rate of mice with systemic *C. albicans* infection. Mice inoculated with PBS served as the vehicle control. (g) Renal pathological analysis of murine subjects 72 h post-systemic infection. Scale bar: 100 µm. (h) Quantification of fungal burden in hepatic and renal tissues 72 h post-systemic infection. The significance levels of *P* values were calculated by comparing with the selected two groups as follows: *0.01 < *P* < 0.05, **0.001 < *P* < 0.01, ***0.0001 < *P* < 0.001 and ****P < 0.0001. The “ns” represents no significant difference. The *n* value in Figure 6f is 15.

(a) Mitochondrial morphology and location of various strains. For convenient comparison, the images of WT and *num11*Δ/Δ are same as Figure 2a. Scale bar: 10 µm. (b) Representative microscopic images of chitin. Scale bar: 10 µm. (c) Representative microscopic images of β-glucan surface exposed. Cells were cultured overnight in YPD medium and stained with anti-(1,3)-glucan antibody and Cy3-conjugated secondary antibody. Scale bar: 10 µm. (d) Average fluorescent intensity (left) and representative flow cytometry profiles (right) of total amount of chitin assessed by CFW. (e) Average fluorescent intensity (left) and representative flow cytometry profiles (right) of CW β-glucan surface exposed cells were incubated with primary anti-β-glucan antibody and PE-conjugated secondary antibody. The significance levels of *P* values were calculated by comparing with the selected two groups as follows: ****P < 0.0001. The “ns” represents no significant difference.

Subsequently, we utilized a systemic infection model in mice to examine the differences in virulence among the WT, the *num11*Δ/Δ, and the *num11*Δ/*NUM11N* strains (Figure 5e). By the second day post-infection, all mice in the WT group had succumbed, whereas the survival rate in the *num11*Δ/Δ group was 90%. By the ninth day post-infection, all mice in the *num11*Δ/*NUM11N* group had succumbed, while the *num11*Δ/Δ group exhibited a 70% survival rate. Log-rank test analysis indicated that the WT group demonstrated significantly increased virulence compared to the Blank group. Conversely, the *num11*Δ/Δ group exhibited significantly reduced virulence relative to the WT group (Figure 5f). Furthermore, the *num11*Δ/*NUM11N* group demonstrated significantly greater virulence compared to the *num11*Δ/Δ group. H&E staining of kidney sections (Figure 5g) revealed that, in contrast to the Blank group, mice infected with the WT and *num11*Δ/*NUM11N* strains exhibited marked pathological alterations. In contrast, mice infected with the *num11*Δ/Δ strain exhibited partial tissue collapse but no significant inflammatory infiltration, indicating that the deletion of *NUM11* mitigated kidney inflammation in systemically infected mice. Additionally, the fungal load in liver and kidney tissues was significantly reduced in the *num11*Δ/Δ group compared to the WT group. Conversely, the fungal load was significantly higher in the *num11*Δ/*NUM11N* group compared to the *num11*Δ/Δ group, with levels in the liver being restored to those observed in the WT group (Figure 5h). Therefore, based on the results of the above two infection models, we have demonstrated that the loss of Num11 significantly may attenuate the pathogenicity of *C. albicans* in vivo.

### Num11 regulating hyphal formation and β-glucan surface exposed in *C. albicans* are not solely by interacting with Mdm36 and dynein

In *S. cerevisiae*, Num11 can form the MECA complex directly with Mdm36 protein and also directly interacts with dynein protein (Dyn1), thereby influencing the mobility of mitochondria and the nucleus [38]. Consequently, we compared the *num11*Δ/Δ strain with knockout strains encoding these two directly interacting proteins in terms of carbon source utilization characteristics, sensitivity to cell wall stress agents, drug sensitivity, hyphal formation ability, and cell wall composition and structure. The results of the spot assay (Figure S4) revealed significant differences between the *num11*Δ/Δ strain and the *mdm36*Δ/Δ strain in carbon source utilization, sensitivity to cell wall stress agents, and drug sensitivity. Although the *num11*Δ/Δ and *dyn1*Δ/Δ strains exhibit highly similar phenotypic characteristics in terms of utilization of fermentable carbon sources, sensitivity to caspofungin and hydrogen peroxide, as well as tolerance to various metal ions, the *dyn1*Δ/Δ strain displays significant functional defects in the utilization of non-fermentable carbon sources and exhibits marked sensitivity to Amphotericin B and Fluconazole. The MIC test results indicate that the *num11*Δ/Δ and *dyn1*Δ/Δ strains exhibit a high degree of consistency in their sensitivity to caspofungin and hydrogen peroxide, with MIC values of 0.125 μg/mL and 6 mM, respectively. However, the *dyn1*Δ/Δ strain shows higher sensitivity to amphotericin B and fluconazole compared to the *num11*Δ/Δ strain: the MIC values for AMB and Flu in *dyn1*Δ/Δ are 0.025 μg/mL and 0.5 μg/mL, respectively, while those for *num11*Δ/Δ are 0.05 μg/mL and 1 μg/mL (S6 Table). This gradient difference in MIC values is in complete agreement with the phenotypic analysis results from previous spot assays (Figure S4). The mitochondrial staining experiment (Figure 6a) revealed that the mitochondria of both the *mdm36*Δ/Δ strain and the *dyn1*Δ/Δ strain exhibited mild damage compared to the WT, showing similarities. In contrast, the mitochondria of the *num11*Δ/Δ strain displayed the most irregular reticular structure, with some fragments in addition to the reticular pattern. Furthermore, based on quantitative analysis using laser scanning confocal microscopy and flow cytometry (Figure 6b-e), we found that compared to WT and *mdm36*Δ/Δ, the *num11*Δ/Δ and *dyn1*Δ/Δ exhibit a significant cell wall remodeling phenotype which is increased chitin biosynthesis and an elevated level of β-1,3-glucan exposure on the cell wall surface. Notably, although there was no statistical difference in the degree of β-glucan surface exposed between the two groups, the chitin content in the *dyn1*Δ/Δ strain was lower than that in the *num11*Δ/Δ strain. Therefore, after synthesizing the results of MIC tests, spot assays, mitochondrial staining, and analyses of cell wall composition and structural changes, we discovered that the mechanism of action of the Num11 anchor protein in *C. albicans* on mitochondrial function and cell wall synthesis differs from that produced solely through interactions with Mdm36 and Dyn1.

### Loss of Num11 affects the cAMP-PKA and Cdc42-Cek1 pathways

**Figure 7.**
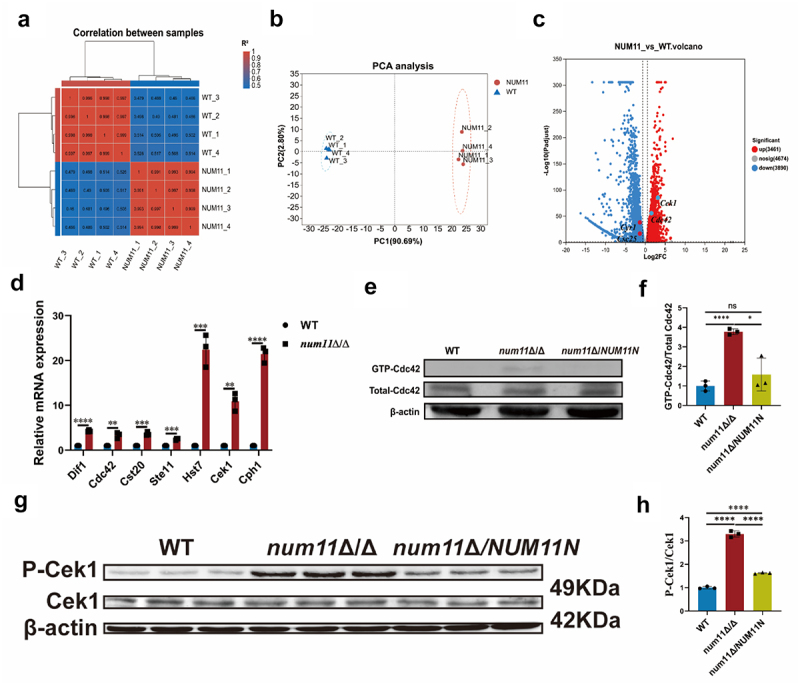
Loss of Num11 can activate Cdc42-Cek1 pathway to induce cell wall re-construction.

To deeply explore the potential molecular mechanisms by which the Num11 regulates the surface exposed of cell wall components, a critical process closely related to the virulence of *C. albicans*, we conducted a detailed comparative transcriptomic analysis between WT strains and *num11*Δ/Δ strains. Subsequently, we performed GO enrichment analysis on the differentially expressed genes between these two types of strains, and the specific results are shown in Figure S5. The most significantly differentially expressed genes are mainly enriched in cytosolic small ribosomal subunits, lipid oxidation, fatty acid oxidation, etc. Later, we conducted Venn analysis between WT strains and *num11*Δ/Δ strains and found that there were 155 differentially expressed genes related to hyphae, 194 differentially expressed genes related to biofilm, and 408 differentially expressed genes related to mitochondria. The results from sample correlation and principal component analysis (Figure 7a,b) revealed substantial transcriptomic differences between the two groups. Subsequent analysis identified that 3,461 genes exhibited significant upregulation, while 3,890 genes demonstrated significant downregulation in the *num11*Δ/Δ knockout strain, encompassing genes located on both homologous chromosomes (Figure 7c).

(a) Correlation analysis of biological reproducibility among samples within each group. Note: WT denotes the WT strain, and NUM11 denotes the *num11*Δ/Δ strain. (b) PCA analysis between WT and *num11*Δ/Δ groups. (c) Volcano plots between WT and *num11*Δ/Δ groups. (d) Gene expression of Cdc42-Cek1 pathway. (e) Pull down test of the activity of Cdc42. (f) The ratio of GTP-bound Cdc42 to total Cdc42 relative to the WT level was determined. (g) Western blot analysis of phosphorylated Cek1 and total Cek1 proteins. (h) The quantification analysis of ratio of phosphorylated Cek1 and total Cek1 proteins. The significance levels of *P* values were calculated by comparing with the selected two groups as follows: * 0.01<*P*<0.05,** 0.001<*P*<0.01,*** 0.0001<*P*<0.001 and **** *P*<0.0001. The “ns” represents no significant difference.

Furthermore, we conducted an in-depth analysis of the specific genes exhibiting altered expression in the *NUM11* deletion strain. Notably, we found a significant upregulation of a series of genes associated with the Cek1 pathway within the MAPK signaling cascade. *C. albicans* activates various signaling pathways in response to environmental changes and stress conditions. Among these, the Cek1 pathway plays a crucial role in transducing numerous environmental signals into the cell, thereby inducing morphological alterations, cell wall polysaccharide synthesis, and β-glucan surface exposed [28]. Subsequently, we sought to validate the upregulation of genes within the Cdc42-Cek1 pathway in the *num11*Δ/Δ strain. Quantitative reverse transcription PCR analysis (Figure 7d) revealed that, in comparison to the WT, the expression levels of *CDC42*, *DFI1*, *CST20*, *STE11*, *HST7*, and *CEK1* genes were significantly elevated in the *num11*Δ/Δ strain, with all differences reaching statistical significance. Subsequently, we investigated the alterations in Cdc42 protein activity in the absence of Num11 protein utilizing a pull-down assay. The assay results revealed a significant increase in the levels of GTP-bound, activated Cdc42 in the *num11*Δ/Δ knockout strain compared to the WT strain. Conversely, the levels of non-activated, GTP-unbound Cdc42 remained largely unchanged (Figure 7e,f). Subsequently, Western blot analyses were conducted utilizing a Phospho-p42/44 antibody to detect the phosphorylated form of Cek1 (Figure 7g). The results indicated that, compared to the WT strain, the expression level of phosphorylated Cek1 protein was elevated in the *num11*Δ/Δ mutant. In contrast, the levels of non-phosphorylated Cek1 remained largely unchanged (Figure 7h). In comparison to the *num11*Δ/Δ strain, the complemented strain exhibited a compensatory trend in the expression levels of both activated protein forms. Therefore, integrating the findings from gene expression, pull-down, and Western blot analyses, we have identified for the first time that the loss of Num1 may activate the Cdc42-Cek1 MAPK metabolic pathway to promote cell wall remodeling and an enhanced surface exposed β-glucan in *C. albicans*.

## Discussion

**Figure 8.**
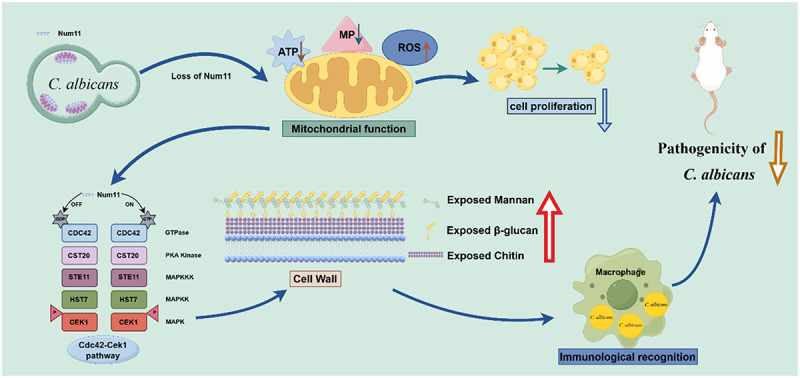
Schematic diagram of the potential mechanism of Num11 affects the pathogenicity of *C. albicans*.

Here, we demonstrate that the C-terminal subcellular localization of the anchoring protein Num11 in *C. albicans* significantly colocalizes with mitochondria and is adjacent to the plasma membrane. The absence of Num11 was found to significantly affect cell division, growth rate, and mitochondrial functions. Specifically, this absence leads to mitochondrial morphological aggregation, reduced ATP production and membrane potential, and increased levels of ROS. Additionally, our research results indicate that Num11 plays a crucial role in regulating cell wall synthesis and surface exposed in *C. albicans*. Upon the loss of Num11, the cells exhibited increased sensitivity to various cell wall-related drugs, accompanied by a significant augmentation in cell wall thickness and the content and surface exposed of β-glucan, chitin, and mannan. This change correlated with increased phagocytosis of *C. albicans* by macrophages and an elevated inflammatory response. Furthermore, experimental studies using mouse systemic infection and *G. mellonella* infection models demonstrated that the absence of Num11 significantly reduced the pathogenicity of *C. albicans* in vivo. Finally, transcriptome analyses suggest that the anchoring protein Num11 influences the surface exposure of cell wall components in *C. albicans* by upregulating the Cdc42-Cek1 signaling pathway. In summary, our experimental results indicate that Num11 plays a crucial role in maintaining mitochondrial function in *C. albicans* to promote the cell proliferation, including the production of suitable ROS, adequate ATP and membrane potential, all of which are achieved through its function of anchoring mitochondria. Conversely, Num11 may regulate the activity of the Cdc42-Cek1 MAPK pathway to modulate the synthesis and surface exposed of polysaccharide components in the cell wall through affection on mitochondria, thereby facilitating the fungal cell ability to conceal surface antigens and evade host immune recognition. Combined these two closely connected potent mechanisms, the anchoring protein Num11 may contribute to the pathogenicity of *C. albicans* (Figure 8).

Firstly, Num11 plays a crucial role in maintaining mitochondrial function in *C. albicans* to promote the cell proliferation by anchoring mitochondria, which is reflected in its impact on the production of suitable ROS, adequate ATP, and membrane potential. Conversely, through affection on mitochondria, Num11 may regulate the synthesis and surface exposed of polysaccharide components in the cell wall by modulating the activity of the Cdc42-Cek1 mitogen-activated protein kinase (MAPK) pathway, thereby enhancing the ability of fungal cells to conceal surface antigens and evade host immune recognition. The working model image was created by Figdraw (https://www.figdraw.com/).

Existing researches have elucidated that mitochondrion participates in the pathogenic process of fungi through multidimensional mechanisms such as metabolite synthesis, membrane construction, drug resistance regulation, and pathogenicity determination [16,40]. Notably, in pathogenic fungi such as *Aspergillus fumigatus* and *Cryptococcus neoformans*, mitochondria enhance host adaptability through mechanisms such as nutrient sensing, stress regulation, and immune evasion [41–43], and the drug-resistant phenotype of *C. albicans* has also been shown to be closely related to mitochondrial functional status [44]. Based on this background, this study systematically explored the regulatory role of Num11 protein on the structure and function of mitochondria in *C. albicans*. Expectedly, the coordinated disruption of mitochondrial membrane potential, intracellular ATP levels, ROS accumulation of *num11*Δ/Δ strain suggest that Num11 may participate in energy metabolism regulation by maintaining mitochondrial homeostasis to regulate the cell growth and other phenotype of *C. albicans*. It is particularly noteworthy that mitochondrial aerobic respiration, as a core aspect of carbon metabolism [45,46], can trigger metabolic reprogramming when its function is impaired. The revealed Num11-mitochondrial regulatory axis provides a new molecular perspective for elucidating fungal environmental adaptation and pathogenic mechanisms. Thus, subsequent research will combine metabolomics and protein interaction network analysis to decipher its action pathways.

Cell wall of fungus, being the outermost component of the cell, facilitates the initial physical interaction between microorganisms and their environment, including the host [19]. Research has unexpectedly shown that mitochondria are essential for proper cell wall development and stability, and they also affect how susceptible fungi are to drugs that target the cell wall [12]. Cell wall masking has been demonstrated to be essential for the pathogenicity of *C. albicans* [47]. Specifically, the surface exposed and concealment of pathogen-associated molecular pattern molecules, such as β-glucan, on the cell wall surface are critical determinants of the virulence of *C. albicans*. Although the interplay between cell wall integrity and mitochondrial homeostasis remains poorly characterized, accumulating evidence indicates that mitochondrial dysfunction in fungal cells correlates with compromised cell wall architecture [13]. Additionally, pharmacological inhibition of the respiratory chain can result in the surface exposed of cell wall polysaccharide components without significantly affecting their synthesis. However, to the best of our knowledge, there are no reports investigating whether anchoring proteins, such as Num1 in *S. cerevisiae*, play a role in regulating the synthesis and surface exposed of cell wall polysaccharide components. Building upon the aforementioned research and inquiries, our objective is to elucidate the alterations in the composition and surface exposed of the *C. albicans* cell wall subsequent to the deletion of the anchoring protein Num11. By examining the cell wall morphology of both the WT strain and the *num11*Δ/Δ strain through transmission electron microscopy and quantifying their respective thicknesses, we observed a significant increase in cell wall thickness in the *num11*Δ/Δ strain compared to the parental strain. These findings substantiate the role of the *NUM11* gene in modulating cell wall morphology. Furthermore, the loss of the Num11 protein in *C. albicans* resulted in a marked increase in sensitivity to cell wall-related drugs and antimicrobials, while no significant change was observed in sensitivity to membrane-associated antifungal agent, fluconazole. Additionally, there was a notable increase in the content and surface exposed of polysaccharide components, such as β-glucan and chitin in the *num11*Δ/Δ strain. Moreover, human and mouse macrophage cell lines demonstrated significantly enhanced recognition and phagocytosis of the knockout strain. These findings suggest that the loss of Num11 could affects the host and *C. albicans* interaction by enhancement of immune recognition of surface exposed of cell wall component.

Subsequently, we investigated and corroborated alterations in pathways associated with the biosynthesis and presentation of cell wall polysaccharide components following the deletion of *NUM11*, utilizing transcriptome analyses. Notably, our findings revealed a significant upregulation of the Cek1 MAPK pathway in *C. albicans*. Additionally, Western blot analysis confirmed a marked increase in the expression level of phosphorylated Cek1 in the *num11*Δ/Δ strain. In unicellular yeasts, the regulation of polarized growth is intricately controlled by Rho GTPases, both spatially and temporally, with Cdc42 serving as the most thoroughly examined example of these enzymes [48]. Notably, Cdc42 is indispensable for the formation of buds during cell division in S. cerevisiae. Furthermore, in the opportunistic fungal pathogen *C. albicans*, Cdc42 holds a prominent position in the biosynthesis and subsequent surface exposed of β-glucan, a fundamental constituent of the cell wall [26,28]. Here, we discovered that a significant upregulation of the active of GTP-bound form of the RHO GTPase protein Cdc42 which functions upstream of Cek1. These findings prompted us to hypothesize that the deletion of *NUM11* may lead to the activation of Cdc42, thereby triggering the Cek1 pathway to result in a pronounced increase in the biosynthesis and surface exposed of cell wall polysaccharides, particularly β-glucan, in *C. albicans*.

Utilizing two in vivo infection models, our observations indicate a significant reduction in the virulence of *C. albicans* following the deletion of the anchoring protein Num11 in both experimental frameworks. Specifically, in the systemic infection model, mice infected with the *num11*Δ/Δ strain exhibited improved overall health and demonstrated significantly extended survival times. Pathological analyses indicated that although fungal burdens were detected in the liver and kidney tissues of mice infected with the *num11*Δ/Δ strain, the extent of renal damage was notably less severe in comparison to the WT and *num11*Δ/*NUM11N* groups. This attenuated virulence is likely due to the enhanced surface exposed of β-glucan and attenuated growth in the *NUM11* knockout strain, which increases its susceptibility to phagocytosis by macrophages in the host.

Most of the MECA-related researches are performed in *S. cerevisiae* [38]. The anchoring protein Num1 of *S. cerevisiae* functions as a multifunctional entity, facilitating the tethering of mitochondria to the plasma membrane during nuclear inheritance and anchoring dynein to the cell cortex. Omer and colleagues proposed a hypothesis that two distinct populations of Num1 clusters, differing morphologically and functionally (namely, small and large clusters), facilitate spindle pulling and mitochondrial-tethering activities at the cell cortex [49]. The large cluster of MECA is composed of Num1 and Mdm36, while the small cluster is composed of Num1 alone. During the cell cycle, the Num1 EF hand domain facilitates the hierarchical integration of organelle positioning pathways, ensuring a regulated order of organelle inheritance [50]. Most recently, Casler and colleagues discovered that the MECA core component, Num1, regulates the distribution of PI(4)P and mitochondrial dynamics through Num1–Scs2 interaction [51]. Consistently, these may explain that why Num11 also influences many mitochondria and other physiological functions in *C. albicans*. The extent to which Num1’s influence on hyphal formation and cell wall composition in *C. albicans* is independently attributable to these distinct roles or is interconnected remains to be elucidated through further investigation. Since our research has identified the Num11 protein as a regulator of Cdc42-Cek1 MAPK pathway activity, investigating the mechanisms of whether Num11 regulates Cdc42 through direct interaction or by modulating the activity of its guanine nucleotide exchange factors and GTPase-activating proteins constitutes a promising avenue for further research.

NuMA serves as a conserved regulatory factor that plays a pivotal role in spindle orientation across various cell types, including epidermal progenitor cells and neural progenitor cells in mammals, neuroblasts in *Drosophila*, and zygotic dividing cells in *Caenorhabditis elegans* [52–54]. Studies on the loss of function of these proteins have demonstrated that NuMA is crucial for the correct orientation of the spindle and the generation of force [52–54]. Notably, the Num11 protein does not have a direct homolog in humans or other mammals and shows only partial functional and motif similarities with the NuMA protein and is primarily found in other pathogenic fungi like *Candida auris*. Therefore, investigation on the developing specific targeted drugs could lead to promising research and development opportunities in clinic. Due to lack of protein structure of Num1, it is also worth to investigate the detail protein structure of Num1 in *S. cerevisiae* and Num11 in *C. albicans.*

In conclusion, our findings suggest that the anchoring protein Num11 may influence the pathogenicity of *C. albicans* in two ways. First, it may generate sufficient energy through mitochondria to regulate fungal cell growth. Second, it may modulate the Cdc42-Cek1 MAPK pathway to control the synthesis and surface exposure of cell wall polysaccharides through mitochondria. Thus, this study will clarify the role of the Num11 in the virulence of *C. albicans* and its potential link to pathogenic processes along this mitochondria-to-cell wall communication axis.

## Materials and methods

### *C. albicans* strains and mediums

The strains utilized in this study are detailed in S1 Table. The *C. albicans’* wild-type strain, SN152, and plasmids, pSN40 and pSN52, were kindly provided by Professor Yuan-Ying Jiang from the New Drug Research Center, School of Pharmacy, Second Military Medical University; plasmid CIp30 was kindly provided by Professor Alistair J.P. Brown (University of St. Andrews); *Escherichia coli* strain DH5α was purchased from Sangon Biotech (Shanghai) Co., Ltd.; other strains were constructed in our laboratory. For routine growth and reproduction, *C. albicans* strains were cultured in yeast extract peptone dextrose (YPD) liquid medium, composed of 1% yeast extract, 2% peptone, and 2% glucose, as well as in YPD agar medium, which includes 1% yeast extract, 2% peptone, 2% glucose, and 2% agar, at 30°C. The strains were preserved in 50% glycerol at −80°C and were retrieved by incubation on YPD solid plates at 30°C.

### Strain construction

*C. albicans* SN152 is a parental WT strain characterized by deficiencies in the leucine (LEU), histidine (HIS), and arginine (ARG) genes [55]. In this study, the *HIS1-LEU2-ARG4* auxotrophic method, combined with the principle of homologous recombination, was employed to construct *C. albicans num11*Δ/Δ, *dyn1*Δ/Δ, and *mdm36*Δ/Δ strains. All primers utilized in the strain construction are detailed in S2 Table, while all plasmids are summarized in S3 Table. Initially, the heterologous selection markers *Candida maltosa LEU2* and *Candida dubliniensis HIS1*, along with the upstream and downstream gene fragments of *NUM11*, were integrated using fusion PCR. The fused DNA fragments containing two selection markers were sequentially transformed into the SN152 strain to generate the *C. albicans NUM11* gene knockout strain. The successful construction of this strain was confirmed via nested PCR. Due to the too long sequence of *NUM11*, ectopic complementation was employed to integrate the N-terminal sequence of *NUM11* gene into the RPS1 locus of the plasmid through homologous recombination. This process utilized a plasmid harboring the heterologous selection marker *C. dubliniensis ARG4* and the CIP30-*NUM11* promoter. Following digestion with Stu I, the construct was transformed into *C. albicans* [55]. The successfully constructed strains *num11*Δ::*LEU2*, *num11*Δ/Δ, *num11*Δ/*NUM11N*, along with the parental strain SN152, were inoculated into distinct auxotrophic media: SC-His, SC-Leu-His, and SC-Leu-His-Arg, with YPD serving as the control. Following a 48-h incubation period at 30°C, growth was subsequently assessed.

By using homologous recombination, specific homologous arms targeting the downstream region of the stop codon of the *NUM11* gene were designed to construct a recombinant vector carrying the mNeonGreen fluorescent protein coding sequence and a selection marker. After transformation into SN152, resistance screening and PCR verification were performed to achieve precise insertion and stable expression of mNeonGreen at the C-terminus of the Num11.

### Subcellular colocalization analysis of Num11 protein with the plasma membrane

To verify the subcellular localization characteristics of the Num11 protein, *C. albicans* WT strain carrying the *NUM11*-mNeonGreen fusion tag were inoculated into YPD liquid medium and cultured with shaking at 200 rpm at 30°C until they reached the logarithmic growth phase (OD600 = 0.8). The cells were washed using a PBS buffer through gradient centrifugation (1007 g, 5 min, repeated twice), and the concentration of the fungal cell suspension was adjusted to 1 × 10^6^ cells/mL using a hemocytometer. Base on previous research [56], the cells were stained in the dark with 5 μM of the plasma membrane-specific fluorescent probe PM-1 (1449483–78–6, MedChemExpress, USA) for 20 min, fluorescent expression was observed using a Stellaris 5 Cryo (Leica, Wetzlar, Germany).

### Mitochondrial morphology observation

To examine mitochondrial morphology, Mito-Tracker Red fluorescent dye was employed [57,58]. Firstly, WT, *num11*Δ/Δ, *num11*Δ/*NUM11N*, *mdm36*Δ/Δ, and *dyn1*Δ/Δ strains were cultured overnight in YPD liquid medium at 30°C with shaking at 200 rpm. Subsequently, the cell concentration was adjusted to 1.0 × 10^6^ cells/mL using sterile PBS. A 200 μM Mito-Tracker Deep Red FM (C1049B, Beyotime, Shanghai, China) stock solution was then added to the cell suspension at a ratio of 1:1000, followed by incubation at 30°C in the dark for 30 min. Following two washes with sterile PBS, mitochondrial morphology was observed at an excitation wavelength of 579 nm.

### Intracellular ATP content measurement

The intracellular ATP content of each strain was quantified utilizing the BacTiter-Glo™ (G8230, Promega, Beijing, China) microbial cell viability assay kit [59]. After overnight cultivation in YPD liquid medium at 30°C with shaking at 200 rpm, the concentrations of the WT, *num11*Δ/Δ, and *num11*Δ/*NUM11N* strains were standardized to 1.0 × 10^7^ cells/mL using sterile PBS. An equivalent volume of the cell suspension was incubated with the BacTiter-Glo reagent for 5 min, after which the relative luminescence unit fluorescence signal was measured using a full-wavelength multifunctional microplate reader.

### Mitochondrial membrane potential measurement

The mitochondrial membrane potential was assessed utilizing the JC-1 (C2003S, Beyotime, Shanghai, China) mitochondrial membrane potential assay kit. After overnight cultivation in YPD liquid medium at 30°C with shaking at 200 rpm, the concentrations of the WT, *num11*Δ/Δ, and *num11*Δ/*NUM11N* strains were standardized to 1.0 × 10^7^ cells/mL using sterile PBS. An equal volume of the cell suspension was then combined with the JC-1 staining working solution and incubated at 37°C in the dark for 20 min. Following two washes with sterile PBS, the fluorescence intensities at emission wavelengths of 590 nm and 530 nm were concurrently measured using a flow cytometer. CCCP serves as a positive control for inducing the decrease of mitochondrial membrane potential. The mitochondrial membrane potential is directly proportional to the ratio of the red fluorescence intensity to the green fluorescence intensity (ratio of FI) [60].

### Intracellular ROS content measurement

The intracellular reactive oxygen species (ROS) levels of each strain were quantified utilizing a ROS assay kit (E004–1–1, Njjcbio, Nanjing, China). After overnight cultivation in YPD liquid medium at 30°C with shaking at 200 rpm, the concentrations of the WT, *num11*Δ/Δ, and *num11*Δ/*NUM11N* strains were standardized to 1.0 × 10^6^ cells/mL using sterile PBS. Subsequently, 2,7-dichlorofluorescin diacetate (DCFH-DA) was introduced to the cell suspension to achieve a final concentration of 10 μM. The mixture was then vortexed and incubated at 30°C in the absence of light for 1 h. Following two washes with sterile PBS, images were acquired using an excitation wavelength of 488 nm and an emission wavelength of 525 nm, maintaining a consistent exposure time. The images were subsequently processed, and fluorescence intensity was quantified using Image J software [59].

### Spot assay

To assess the growth dynamics of various strains under different stress conditions, WT, *num11*Δ/Δ, *num11*Δ/*NUM11N*, *mdm36*Δ/Δ, and *dyn1*Δ/Δ strains were initially cultured overnight in YPD liquid medium at 30°C with agitation at 200 rpm. Subsequently, the cell concentration was standardized to 1.0 × 10^6^ cells/mL using sterile PBS and further subjected to a tenfold dilution to achieve a final concentration of 1.0 × 10 [2] cells/mL. Five microliters of the diluted cell suspension were inoculated onto agar plates containing either fermentable carbon sources (2% glucose, 2% maltose) or non-fermentable carbon sources (2% ethanol, 2% citrate, 2% glycerol), as well as various pharmacological agents including 2 μg/mL fluconazole (Flu) (155347–36–7 Shyuanye, Shanghai, China), 0.5 μg/mL caspofungin (CAS) (1202167–57–4, Shyuanye, Shanghai, China), 0.05 μg/mL Amphotericin B (AmB) (1397–89–3, Shyuanye, Shanghai, China) [61], and cell wall stress-inducing components (50 μg/mL CR (573–58–0, Shyuanye, Shanghai, China), and 200 μg/mL CFW (18909–100 ML-F, Sigma, USA)) [62]. The growth of each strain was subsequently monitored.

### Colony size measurement

After overnight cultivation in YPD liquid medium at 30°C with shaking at 200 rpm, the concentrations of the WT, *num11*Δ/Δ, and *num11*Δ/*NUM11N* strains were standardized to 300 cells/mL using sterile phosphate-buffered saline. Subsequently, the cell suspension was spread onto YPD agar plates and incubated in an inverted position at 35°C for 48 h. The dimensions of individual colonies were measured using a vernier caliper and documented according to previous research [37].

### Growth curve determination

To determine the growth curve, the strains of WT, *num11*Δ/Δ, and *num11*Δ/*NUM11N* were cultured overnight in YPD liquid medium at 30°C with agitation at 200 rpm to ensure they reached the exponential growth phase. Subsequently, the cells were washed twice with sterile PBS and resuspended in Sabouraud dextrose broth to an optical density (OD) of 0.04 at 600 nm. The cultures were then incubated at 30°C with agitation at 200 rpm. The OD_600_ value of each culture was measured at 2-h intervals, and these values were plotted to generate the growth curve for each strain.

### Determination of minimum inhibitory concentration

According to the CLSI M27-M44S protocol established by the Clinical and Laboratory Standards Institute (CLSI) [63], the broth microdilution method in 96-well plates was used to determine the Minimum Inhibitory Concentration (MIC) of the WT, *num11*Δ/Δ, *mdm36*Δ/Δ, and *dyn1*Δ/Δ strains against Flu, CAS, AmB, hydrogen peroxide (H₂O₂), and dimethyl sulphoxide (DMSO). In this experiment, RPMI-1640 liquid medium was used to adjust the concentration of each fungal suspension to 2 × 10 [3] cells/mL. One hundred microliters of each drug solution, after serial dilution, was added to the 96-well plate, followed by 100 μL of fungal suspension in each well. A column of blank controls was set up by adding 200 μL of RPMI-1640 liquid medium. The plates were incubated statically at 37°C for 48 h. The experiment was repeated three times.

### RNA-Seq analysis

Total RNA for transcriptome sequencing was extracted from both WT (*n* = 4) and *num11*Δ/Δ (*n* = 4) strains of *C. albicans*. The concentration, purity, and integrity of the extracted RNA were assessed using a Nanodrop2000 spectrophotometer and agarose gel electrophoresis. mRNA was isolated from the total RNA by exploiting A-T base pairing with poly-A tails using magnetic beads conjugated with oligo (dT). The isolated mRNA was then subjected to fragmentation, producing small fragments of approximately 300 base pairs, which were subsequently reverse-transcribed into complementary DNA (cDNA). Following the establishment of a stable double-stranded structure, the adaptor was ligated. Sequencing was subsequently performed by Majorbio in China. Genes exhibiting a |log_2_FC| greater than 1 and a p-value less than 0.05 were selected for further analysis. To interpret the gene expression data, gene set cluster analysis, sample relationship analysis, and differential expression analysis were conducted base on previous research [64].

### Quantitative real-time PCR

After culturing the strains of WT, *num11*Δ/Δ, and *num11*Δ/*NUM11N* overnight in YPD liquid medium at 30°C with agitation at 200 rpm, the fungal suspension was transferred to fresh YPD liquid medium and further cultured until it reached the logarithmic growth phase (with an OD600 value of 0.5–0.8). RNA was subsequently extracted using the Sijie RNA Extraction Kit and reverse transcribed into cDNA utilizing the ToloScript ALL-in-one RT EasyMix (22106, Tolobio, Shanghai, China) for qPCR Kit. Quantitative reverse transcription PCR (RT-qPCR) was then conducted employing the 2×Q3 SYBR qPCR Master Mix Kit (22201, Tolobio, Shanghai, China), with the synthesized cDNA serving as the template. *β-actin* gene served as the internal reference standard, and the relative quantification of gene expression was determined using the 2^−ΔΔCT^ method [65]. The primers employed for the RT-qPCR assays are detailed in Supplementary S4 Table.

### Transmission electron microscopy observation of cell wall morphology

The strains of WT and *num11*Δ/Δ were cultivated overnight in YPD liquid medium at 30°C with agitation at 200 rpm., the cells were washed twice with sterile PBS. Subsequently, the cells were immersed in 2.5% glutaraldehyde, resuspended in PBS buffer, and fixed with 1% osmium tetroxide. Dehydration was achieved through a graded ethanol series (30%, 50%, 80%, 95%, 100%) and 70% ethanol uranyl acetate. The cells were then infiltrated with a propylene oxide: epoxy resin mixture (1:1) followed by pure epoxy resin, before being embedded in pure epoxy resin. Ultrathin sections were prepared, mounted on copper grids, and subjected to electron staining.

### Measurement of β-glucan and mannan content in cell wall

Based on previous research [66–68], to quantify the β-glucan and mannan content within the cell wall, the strains of WT, *num11*Δ/Δ, and *num11*Δ/*NUM11N* were cultivated overnight in 100 milliliters of fresh YPD liquid medium at 30°C and 200 rpm. The resulting cell biomass was harvested, washed twice with sterile water, and subsequently freeze-dried. Glass beads with diameters ranging from 0.4 to 0.6 mm were introduced, and the cells were lysed using a tissue grinder, performing 15 cycles of 60 s each, with 1-min intervals on ice. The lysed cells underwent five washes with 1 M NaCl (7647–14–5, Damao, Tianjin, China), Subsequently, the samples were boiled at 100°C for 10 min in an extraction buffer (pH 8.0) consisting of 50 mM Tris-HCl (BMB3349, Bomei, Hefei, China), 2% sodium dodecyl sulphate (SDS, 151–21–3, Sigma, USA), 0.3 M β-mercutoethanol (QM7141, Bomei, Hefei, China), and 1 mM EDTA (YE2154, Bomei, Hefei, China). Subsequently, the disrupted cell walls were washed three times with sterile water, freeze-dried, and further processed. A sample of 3 mg of the cell wall material was then weighed and suspended in 1 mL of distilled water. The mixture underwent sonication for 10 min, followed by centrifugation at 1007 g for 30 min. The supernatant was subsequently collected and utilized for the detection of mannan content in the cell wall via a Mannan ELISA detection kit (EY-L0011, Guyan, Shanghai, China). For the isolation of β-glucan, 0.5 mg of dry cell wall material was treated with 500 μL of 0.7 M NaOH (1310–73–2, Sinopharm, Shanghai, China) at 75°C for 60 min to facilitate alkaline extraction. The insoluble precipitate, primarily composed of β-glucan, was isolated by centrifugation at 1678 g for 5 min, followed by two additional rounds of alkaline extraction. The resultant precipitate was then freeze-dried and stored at −20°C. For cell wall hydrolysis, 1 mL of 2 M trifluoroacetic acid (ST0781, 100%, Bomei, Hefei, China) was added to the sample, and the mixture was heated at 100°C for 3 h. Subsequently, the TFA was evaporated in a water bath maintained at 90°C for 1 h. The β-glucan content of each strain was quantified using a glucose assay kit (E1010, APPLYGEN, Beijing, China), as described in references.

### Measurement of chitin, surface exposed β-glucan, mannan, and chitin content in the cell wall

Based on previous research [39,69], to quantify the levels of chitin, surface-exposed β-glucan, mannan, and chitin within the cell wall, strains WT, *num11*Δ/Δ, and *num11*Δ/*NUM11N* were cultivated overnight in 6 mL of fresh YPD liquid medium at 30°C with agitation at 200 rpm. Subsequently, the fungal concentration was standardized to 1.0 × 10^6^ cells/mL using sterile PBS. The systematic analysis of fungal cell wall components was performed using flow cytometry (BD-FACS Celesta) combined with fluorescent probe technology: (1) Chitin content analysis: Cells were stained with 500 μL Calcofluor White (10 μg/mL 18,909–100 ML-F, Sigma, USA) for 5–10 min at room temperature, washed three times with PBS, and total chitin content was detected through the FL1 channel (Ex/Em = 488/570 nm); (2) β-Glucan surface exposed analysis: After blocking with 3% bovine serum albumin (BSA, 9048–46–8, BioFRoxx, Shanghai, China), cells were sequentially incubated with a monoclonal anti-β-glucan antibody (1:300, 400–2, Bioscience Supplies, Australia) for 1.5 h at 4°C, followed by Cy3-labeled goat anti-mouse IgG (1:100, A22210, Abbkine, Shanghai, China) for 20 min at 4°C, with quantification through the FL2 channel (Ex/Em = 488/570 nm); (3) Chitin surface exposed analysis: Cells were labeled with 500 μL Alexa Fluor 488-conjugated wheat germ agglutinin (WGA, 10 μg/mL, W11261, Invitrogen, California, USA) for 30 min at 4°C in the dark, and analyzed through the FL1 channel (Ex/Em = 488/520 nm); (4) Mannan surface exposed analysis: Cells were stained with 500 μL Concanavalin A conjugates (Con A, 50 μg/mL, C21421, Invitrogen, California, USA) for 30 min at 4°C and detected through the FL4 channel (Ex/Em = 650/665 nm). Critical steps included PBS washing (1007 g × 5 min) and negative controls. All experiments analyzed 10,000 cells, with fluorescence intensity data processed using FlowJo V10.

### Macrophage phagocytosis of *C. albicans*

The phagocytosis was monitored in RAW 264.7 and THP-1 purchased from Shanghai Cell Bank of the Chinese Academy of Sciences (Shanghai, China) based on the existing steps with fewer modifications [22,70]. RAW 264.7 macrophages and THP-1 monocytes were maintained at 37°C with 5% CO₂ in complete growth media: DMEM (CA002-500 mL, SparkJade) for RAW 264.7 and RPMI-1640 (CF0001-500 mL, SparkJade) for THP-1, both supplemented with 10% FBS (KY-01000S, Kangyuan Biologicals) and 1% penicillin–streptomycin (C0222, Beyotime). Cells were seeded at initial densities of 1 × 10^5^ cells/mL (RAW 264.7) and 8 × 10^5^ cells/mL (THP-1 pre-stimulated with 1 μg/mL TPA (GN10444, GLPBIO)), respectively, followed by overnight culture to achieve optimal confluency prior to supernatant removal. WT, *num11*Δ/Δ, and *num11*Δ/*NUM11N* fungal suspensions were added to the macrophages at a ratio of 3:1 (*C. albicans* to macrophages) and co-cultured for 3 h. Following the initial procedure, the medium was discarded, and the cells were fixed overnight at 4°C using 4% paraformaldehyde (143174, Biosharp, Shanghai, China). The cells were subsequently washed three times with PBS and blocked with 5% goat serum (SBJ-SE-GO012, SenBeiJia, Nanjing, China) at room temperature for 30 min. After proper washes with PBS, the rabbit polyclonal FITC-conjugated anti-*C. albicans* antibody (1:250, AB21164, Abcam, Cambridge, UK) diluted in PBS containing 1% bovine serum albumin (BSA, 9048–46–8, Yien Chemical Technology Co., Ltd, Shanghai, China) was added to stain the fungal cells which were not phagocytosed. Post 3 h of incubation, the fungal cells were washed with PBS containing 0.05% BSA and 0.05% Tween 20 (9005–64–5, HuaxiangM Kejie Biotechnology Co., Ltd, Hubei, China) for three times. Finally, the samples were examined and imaged using an inverted fluorescence microscope.

### Establishment of a *G. mellonella* larvae infection with *C. albicans*

Based on previous research [71], totally 72 *G. mellonella* larvae exhibiting agile movements and a body length of 2.0–2.5 cm were selected to evaluate the impact of the Num11 protein on the virulence of *C. albicans*. *G. mellonella* were maintained at 15°C in the dark to suppress pupation. Larvae weighing 0.22 ± 0.03 g were selected for the experiments, and all procedures were completed within 2 weeks after receipt. The strains of WT, *num11*Δ/Δ, and *num11*Δ/*NUM11N* were cultivated overnight in 100 milliliters of fresh YPD liquid medium at 30°C with agitation at 200 rpm. Subsequently, the fungal suspension was washed twice with PBS and adjusted to a concentration of 8.0 × 10^6^ cells/mL. The larvae were then randomly assigned to one of four groups: Blank group, WT group, *num11*Δ/Δ group, and *num11*Δ/*NUM11* group. A 10 µL aliquot of the respective fungal suspension was administered into the left or right hind leg of the larvae using a precision microinjection needle. Subsequently, the larvae were incubated at 37°C. After a period of 48 h, three larvae from each experimental group were randomly selected, homogenized, and plated on YPD agar plates. These plates were then incubated in an inverted position at 30°C for an additional 48 h to assess fungal burden. Furthermore, the mortality rate of *G. mellonella* larvae was monitored at 24-h intervals to construct a survival curve. Survival analysis was conducted utilizing the Log-rank Test.

### Establishment of a mouse model of systemic infection with *C. albicans*

Based on previous research [72], 60 female Balb/C mice (6–7 weeks old, 20 ± 2 g) were housed under controlled environmental conditions: temperature of 23 ± 2°C, relative humidity of 40–70%, and a 12-h light/dark cycle. The mice were obtained from Hefei Qingyuan Biotechnology Co., Ltd. The mice were provided with standard granulated feed and tap water for a 7-day acclimatization period and were housed in SPF-grade animal rooms with condition of 23 ± 2°C, 40–70% relative humidity, 12-h light/dark cycle. All procedures involving the maintenance and treatment of the animals adhered to the regulations set forth by the Animal Ethics Committee of the Chinese Center for Disease Control and Prevention and followed the “Guidelines for the Care and Use of Laboratory Animals” in China (Animal Ethics Number: AHUCM-mouse -2,022,152). We also adhere to ARRIVE guidelines to perform this animal experiment. In the second week, all mice were randomly and evenly allocated into four groups: PBS group, WT group, *num11*Δ/Δ group, and *num11*Δ/*NUM11N* group, with each group comprising 15 mice. Of these, 5 mice were designated for the assessment of kidney and liver fungal load and pathological section examination, while the remaining 10 mice were used to determine survival percentage. Cyclophosphamide (Cat No. 50–18–0, Aladdin, Shanghai, China) was administered intraperitoneally at a dosage of 150 mg/kg on day 4 prior to infection, and at 100 mg/kg on day −1 and +2 to induce immunosuppression. In accordance with the experimental groups, an adjusted fungal suspension, and an equivalent volume of sterile phosphate-buffered saline (PBS) were administered to the mice via tail vein injection at a dosage of 100 μL per mouse. Commencing from the first day post-modeling, the general condition of the mice was monitored daily, and survival times were documented. Following a 15-day observation period, the survival percentage was calculated, a survival curve was generated, and survival analysis was conducted using the Log-rank Test. Seventy-two hours post-modeling, five mice from each experimental group were euthanized via cervical dislocation. After deep anesthesia was induced by intraperitoneal injection of 3% pentobarbital sodium saline solution (at a dose of 100 mg/kg), cervical dislocation was performed to euthanize the mice. Under sterile conditions, the left kidney was excised and subsequently fixed in 4% paraformaldehyde for a minimum of 24 h. Concurrently, liver tissue and the right kidney were extracted, rinsed with sterile saline, weighed, and homogenized with the addition of 1 mL of sterile phosphate-buffered saline (PBS). The resultant homogenate was then serially diluted into four concentration gradients using sterile PBS at a tenfold dilution ratio. The diluted solutions were subsequently plated on YPD medium supplemented with 100 U/mL penicillin and 0.1 mg/mL streptomycin. After an incubation period of 48 h at 30°C, the resulting colonies were enumerated. The mean colony count was determined and used to calculate the colony-forming units per gram (CFU/g) using the formula: $\mathrm{lo}g_{10}\mathrm{CFU}/g=\mathrm{lo}g_{10}$ (mean colony count from three plates × dilution factor)/organ weight.

### Hematoxylin and eosin staining of mouse tissues

Kidney tissues from each experimental group were subjected to standard hematoxylin and eosin (H&E) staining protocols. The tissues were decolorized using a graded series of ethanol solutions (70%, 80%, and 90%) followed by xylene, and subsequently embedded in paraffin. Transverse sections were then prepared to yield final tissue slices of 4 µm thickness, which were mounted on glass slides. Following H&E staining results, tissue damage was assessed microscopically [73].

### Protein extraction and Western blot

Based on previous research [74], the strains of WT, *num11*Δ/Δ, and *num11*Δ/*NUM11N* were cultivated overnight in 100 milliliters of fresh YPD liquid medium at 30°C with agitation at 200 rpm. Subsequently, the cells were washed twice with sterile PBS. Approximately 10 mg of fungal cells were transferred to 1.5 mL Eppendorf tubes containing 1 mL of lysis buffer and mixed thoroughly by inversion. Following this, 150 µL of Trichloroacetic acid (TCA, 76–03–9, Merck, Germany) was added, the mixture was vortexed for 30 s, and then incubated on ice for 10 min. The mixture was subjected to centrifugation at 4364 g and 4°C for 5 min, after which the supernatant was discarded. Subsequently, 100 µL of 1 M Tris and 100 µL of 2× SDS sample buffer were added to the precipitate. The resulting mixture was heated to 95°C for 5 min with intermittent vortexing three times until the precipitate was fully dissolved. A final centrifugation step was conducted at 4364 g for 1 min. Phosphorylated Cek1 was identified utilizing the anti-phospho-p44/42 MAPK antibody (catalog no. 4370; Cell Signaling Technology, Danvers, MA, USA). Total Cek1 was identified utilizing the anti-ERK1/2 Rabbit Polyclonal Antibody (AB2014, Beyotime, Shanghai, China). Detection of β-actin was performed with an anti-β-actin antibody (Zenbio, Chengdu, China). Subsequently, blot images were acquired using the Image Quant LAS 4000 biomolecular imager.

### Pulldown assay for active GTPases

Based on previous research [28], the strains of WT, *num11*Δ/Δ, and *num11*Δ/*NUM11N* were cultivated overnight in 100 milliliters of fresh YPD liquid medium at 30°C with agitation at 200 rpm. Following cultivation, the cells were washed twice with PBS. Subsequently, glass beads with diameters ranging from 0.4 to 0.6 mm were introduced, and the cells were lysed using a tissue grinder for 10 cycles, each cycle lasting 60 s with a 10-s immersion in liquid nitrogen between cycles. The cells were then resuspended in 1×Assay/Lysis buffer and subjected to repeated pipetting to ensure complete lysis. The resulting cell lysate was transferred to an appropriate tube and incubated on ice for 30 min. Centrifugation was conducted at 4028 g and 4°C for a duration of 10 min. The resulting supernatant, containing approximately 1–2 mg of total protein, was collected, and maintained on ice for subsequent use. Active Cdc42 was then affinity-precipitated utilizing the Cdc42 Pulldown Activation Assay Kit (80701, Wuhan Newest Biotechnology Co., Ltd.). Detection of β-actin was achieved using an anti-β-actin antibody (Zenbio, Chengdu, China). Finally, blot images were acquired using the ImageQuant LAS 4000 biomolecular imager.

### Statistical analysis

GraphPad Prism 6.02 was utilized for conducting the statistical analysis. The information is provided from a minimum of three distinct trials and represented as the average ± standard deviation (SD) (*n* ≥ 3). To compare two groups of data, the Student's t-test was employed. Additionally, for comparing multiple groups of data, the ANOVA test was applied. Significant differences were considered for *P*-values below 0.05.

## Supplementary Material

Table S1.docx

S3 Fig.docx

Table S2.docx

S5 Fig.docx

Table S5.xlsx

S2 Fig.docx

S1 Fig.docx

Table S4.docx

Table S6.docx

Table S3.docx

S4 Fig.docx

## Data Availability

Raw data are available via Mendeley Data (DOI: 10.17632/j599779dd2.2). Supplementary materials are available via Figshare (DOI: 10.6084/m9.figshare.28740110).
