# Supplementary material for: Mitochondrial anchor protein Num11 is key to pathogenicity of Candida albicans by affecting mitochondrial function and cell wall masking
Source: Virulence. 2025 Jun 18;16(1):2519149. doi: 10.1080/21505594.2025.2519149 (PMC12184122; doi:10.1080/21505594.2025.2519149)
Supplement: Table S1.docx [file KVIR_A_2519149_SM4326.docx]

**Table S1. *Candida albicans* strains used in this study**

| Strain | Genotype^*^ | Parent | Reference |
| --- | --- | --- | --- |
| SN152 (wild type strain) | *arg4*Δ*/arg4*Δ *leu2*Δ*/leu2*Δ *his1*Δ*/his1*Δ *URA3/ura3*Δ∷*imm^434^ IRO1/iro1*Δ∷*imm^434^* | SC5314 | Noble^1^ |
| *num11*Δ∷*LEU2* | *NUM11/num11*Δ∷*C.m.LEU2 arg4*Δ*/arg4*Δ *leu2*Δ*/leu2*Δ *his1*Δ*/his1*Δ *URA3/ura3*Δ∷*imm^434^ IRO1/iro1*Δ∷*imm^434^* | SN152 (wild type strain) | This study |
| *num11*Δ/Δ | *num11*Δ∷*C.m.LEU2/num11*Δ∷*C.d.HIS1 leu2*Δ*/leu2*Δ *his1*Δ*/his1*Δ *URA3/ura3*Δ∷*imm^434^ IRO1/iro1*Δ∷*imm^434^* | SN152 (wild type strain) | This study |
| *dyn1*Δ/Δ | *dyn1*Δ∷*C.m.LEU2/dyn1*Δ∷*C.d.HIS1 leu2*Δ*/leu2*Δ *his1*Δ*/his1*Δ *URA3/ura3*Δ∷*imm^434^ IRO1/iro1*Δ∷*imm^434^* | SN152 (wild type strain) | This study |
| *mdm36*Δ/Δ | *mdm36*Δ∷*C.m.LEU2/mdm361*Δ∷*C.d.HIS1 leu2*Δ*/leu2*Δ *his1*Δ*/his1*Δ *URA3/ura3*Δ∷*imm^434^ IRO1/iro1*Δ∷*imm^434^* | SN152 (wild type strain) | This study |
| *num11*Δ/*NUM11N* | *num11*Δ∷*C.m.LEU2/num11*Δ∷*C.d.HIS1 leu2*Δ*/leu2*Δ *his1*Δ*/his1*Δ *URA3/ura3*Δ∷*imm^434^ IRO1/iro1*Δ∷*imm^434^ RPS1/rps1∷*CIp30*-NUM11N* | *num11*Δ/Δ | This study |
| *NUM11*-mNeonGreen | *arg4*Δ*/arg4*Δ *leu2*Δ*/leu2*Δ *his1*Δ*/his1*Δ *URA3/ura3*Δ∷*imm^434^ IRO1/iro1*Δ∷*imm^434^ NUM11*/*NUM11*::mNeonGreen-NAT | SN152 (wild type strain) | This study |

^*^ *C.m.*, *Candida maltosa*; *C.d.*, *Candida dubliniensis*.

1. Noble SM, Johnson AD. Strains and strategies for large-scale gene deletion studies of the diploid human fungal pathogen *Candida albicans*. *Eukaryot Cell.* 2005;4(2):298-309.
