# Supplementary material for: Mitochondrial anchor protein Num11 is key to pathogenicity of Candida albicans by affecting mitochondrial function and cell wall masking
Source: Virulence. 2025 Jun 18;16(1):2519149. doi: 10.1080/21505594.2025.2519149 (PMC12184122; doi:10.1080/21505594.2025.2519149)
Supplement: S3 Fig.docx [file KVIR_A_2519149_SM4325.docx]

**
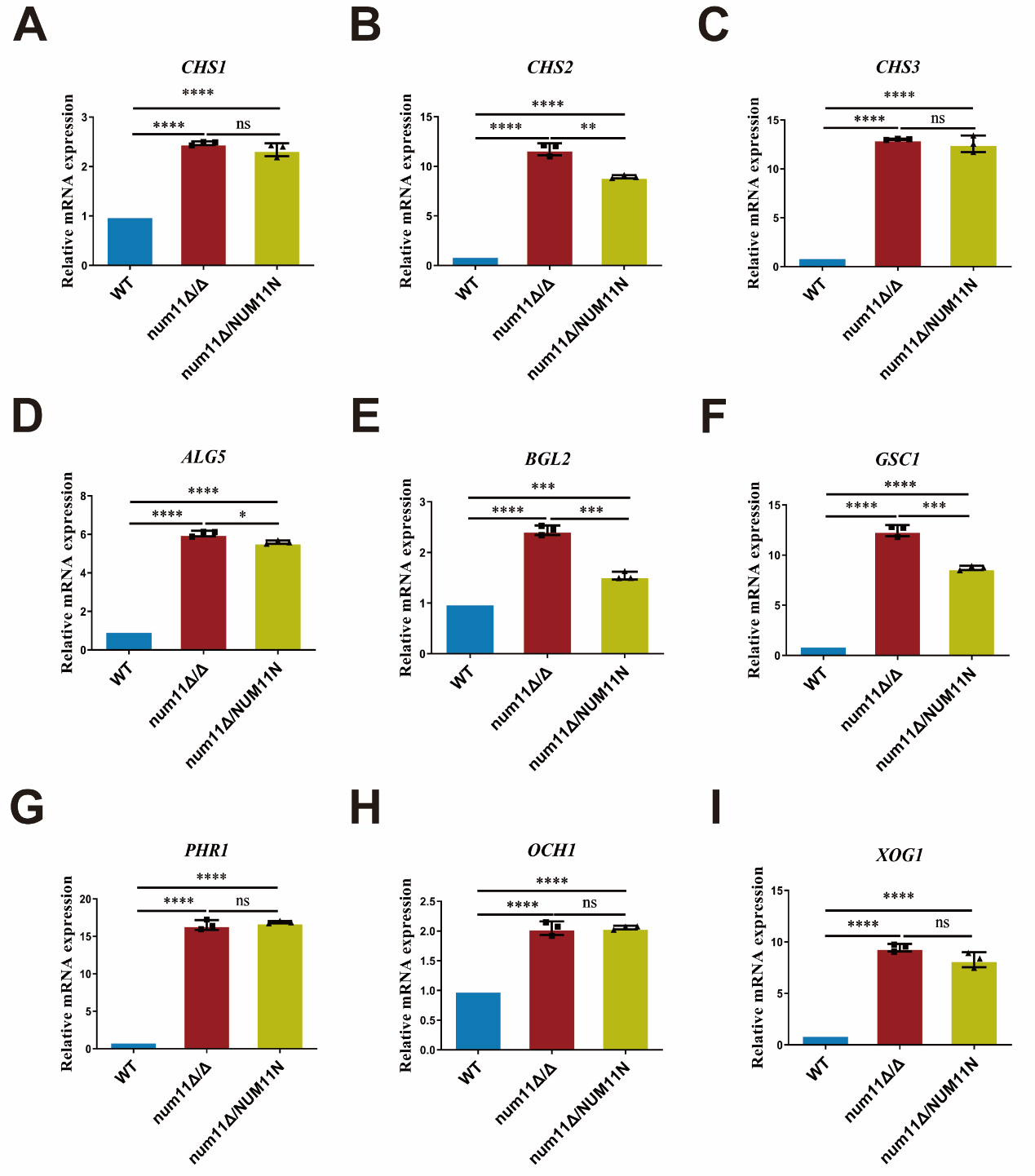
**

**Fig S3. The transcription levels of genes related to cell wall synthesis in each strain.** A - I: Gene expression related to cell wall synthesis. The significance levels of *P* values were calculated by comparing with the WT group as follows: * 0.01≤*P*<0.05,** 0.001<*P*<0.01,*** 0.0001≤*P*<0.001 and **** *P*<0.0001. The“ns”represents no significant difference.
