# Supplementary material for: Mitochondrial anchor protein Num11 is key to pathogenicity of Candida albicans by affecting mitochondrial function and cell wall masking
Source: Virulence. 2025 Jun 18;16(1):2519149. doi: 10.1080/21505594.2025.2519149 (PMC12184122; doi:10.1080/21505594.2025.2519149)
Supplement: Table S2.docx [file KVIR_A_2519149_SM4324.docx]

**Table S2. The primers used in this study.**

| Primer Name | Sequence (5’ to 3’) |
| --- | --- |
| NUM11-2 | ccgctgctaggcgcgccgtgACCAGTGTGATGGATATCTGC |
| NUM11-5 | gcagggatgcggccgctgacAGCTCGGATCCACTAGTAACG |
| NUM11-1 | GCGTACTAATAAAATTAGCAAC |
| NUM11-3 | CACGGCGCGCCTAGCAGCGGTTTCAAAGTAAATGGTATAGGGAT |
| NUM11-4 | GTCAGCGGCCGCATCCCTGCAAAGATTAGTTGAGATGTGA |
| NUM11-6 | TGTTTGCACTTTAGGAA |
| LEU2 Check Left | AGAATTCCCAACTTTGTCTG |
| LEU2 Check Right | AAACTTTGAAC CCGGCTGCG |
| HIS1 Check Left | ATTAGATACGTTGGTGGTTC |
| HIS1 Check Right | AACACAACTGCACAATCTGG |
| NUM11 ORF Left | TATCCATGCCAACGCAAC |
| NUM11 ORF Right | AATCTGTTTCCTTAACGGCTA |
| NUM11 Check Left | AGTTCTAGGATTAATTGCCAT |
| NUM11 Check Right | AATATTGAGAGCTACACGGAA |
| NUM11ReF | TCGATACCGTCGACCATGTTTGACCAAGGTGATGTCTT |
| NUM11ReR | CGGGCCCCCCCTCGATTTGCCTTTGATGTAATGTGTTC |
| Clp30 ReF | TCGAGGGGGGGCCCGGTA |
| Clp30 ReR | GGTCGACGGTATCGATAAGCTTGA |
| Re up Check Left | TCTTCGGAACCTTGTAAGTCG |
| Re up Check Right | TCTGATTAGCCAATTCGGAGT |
| Re down Check Left | TATCCATGCCAACGCAAC |
| NUM11-Tag-P1 | CTGCTGCTATAGTAAGTGTT |
| NUM11-Tag-P2 | AGAATTggtggtagtggtatggtttctaaag |
| NUM11-Tag-P3 | taccactaccaccAATTCTTCTCATCGAC |
| NUM11-Tag-P4 | agcggccgccAAAGATTAGTTGAGATGTG |
| NUM11-Tag-P5 | AGATATATGATTCAAATTCCGTA |
| NUM11 Tag Check Left | AGACCTAAAAGAATTACATCGC |
| NUM11 Tag Check Left | aaataattagcagccattggt |
| DYN1-2 | ccgctgctaggcgcgccgtgCCAGTGTGATGGATATCT |
| DYN1-5 | gcagggatgcggccgctgacAGCTCGGATCCACTAGTAA |
| DYN1-1 | AAATCAAGTAGGTATTGTGC |
| DYN1-3 | CACGGCGCGCCTAGCAGCGGTGTGTTGAATTAAATTTC |
| DYN1-4 | GTCAGCGGCCGCATCCCTGCATTTTTAGTTTATATTTTAATG |
| DYN1-6 | TTCCATAAGCAGCCACGTA |
| DYN1 ORF Left | TCAAGATGTTAATTGCCGAGA |
| DYN1 ORF Right | CTGCGTGTAATAAATGAACCC |
| DYN1 Check Left | CATTGTCATCATCGTCGTC |
| DYN1 Check Right | TTTCAGCTCCTATTACTTGTG |
| MDM36-2 | ccgctgctaggcgcgccgtgATTCATTAAATCTATTACCCTTGC |
| MDM36-5 | gcagggatgcggccgctgacAAGGGGAAACTTCCCGGT |
| MDM36-1 | TTTGTTTGAACCATACGAT |
| MDM36-3 | GCAAGGGTAATAGATTTAATGAAT |
| MDM36-4 | ACCGGGAAGTTTCCCCTT |
| MDM36-6 | CAAGAAATTGTTCACCT |
| MDM36 ORF Left | CATGAAATATCAGAACGACGA |
| MDM36 ORF Right | TTCGGAATACATGTCATCAGG |
| MDM36 Check Left | AACGCATTACATATCCGACCA |
| MDM36 Check Right | TGTCAACAAGATCGCAAG |

All primers were designed by SnapGene Viewer software.
