# Supplementary material for: Mitochondrial anchor protein Num11 is key to pathogenicity of Candida albicans by affecting mitochondrial function and cell wall masking
Source: Virulence. 2025 Jun 18;16(1):2519149. doi: 10.1080/21505594.2025.2519149 (PMC12184122; doi:10.1080/21505594.2025.2519149)
Supplement: S5 Fig.docx [file KVIR_A_2519149_SM4323.docx]

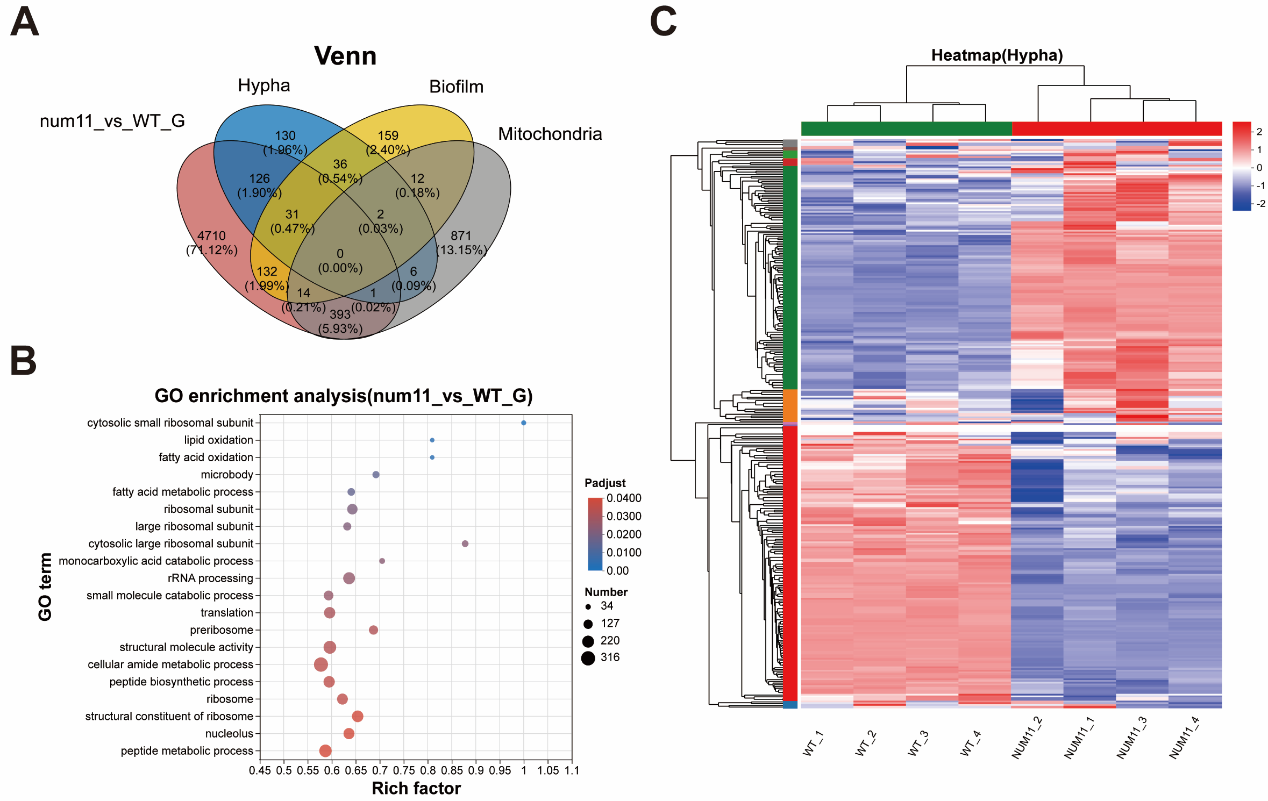


**Fig S5. Transcriptome analysis of various strains.** (A) Venn analysis of differentially expressed gene sets between WT and *num11*Δ/Δ. (B) GO enrichment analysis of differentially expressed genes between WT and *num11*Δ/Δ. (C) Heatmap of clustering analysis of all genes. Note: WT denotes the Wild-Type group, and NUM11 denotes the *num11*Δ/Δ group.
