# Supplementary material for: Mitochondrial anchor protein Num11 is key to pathogenicity of Candida albicans by affecting mitochondrial function and cell wall masking
Source: Virulence. 2025 Jun 18;16(1):2519149. doi: 10.1080/21505594.2025.2519149 (PMC12184122; doi:10.1080/21505594.2025.2519149)
Supplement: S2 Fig.docx [file KVIR_A_2519149_SM4321.docx]

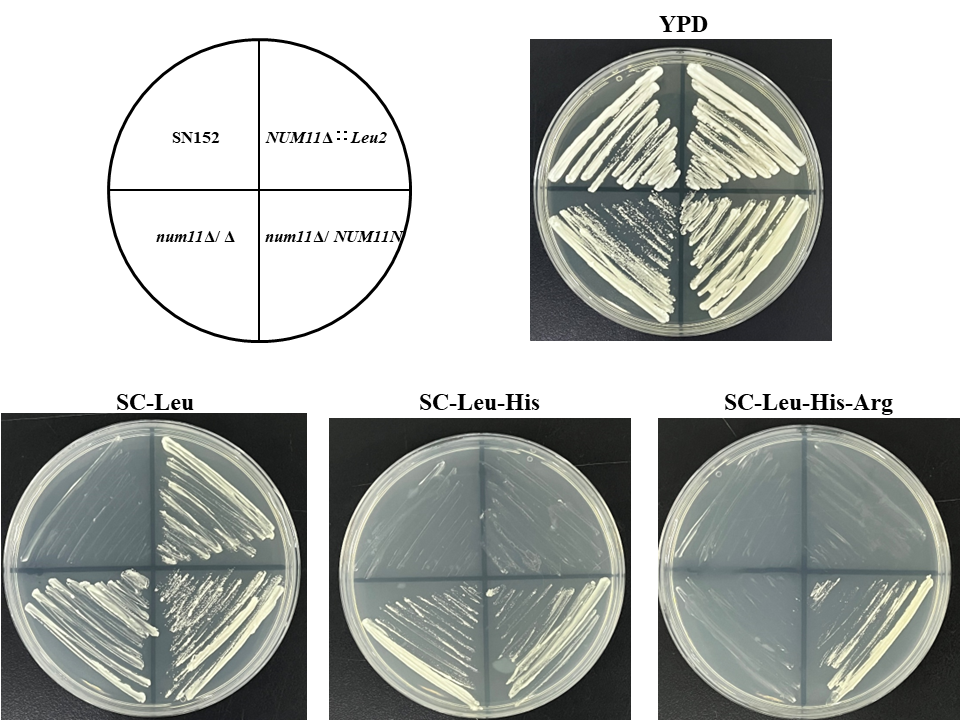


**Fig S2. Growth detection of WT, *num11*Δ∷*LEU2*, *num11*∆/∆, and *num11*Δ/*NUM11*N on YPD, SC-Leu, SC-Leu-His and SC-****Leu-His-Arg media.** The wild-type (SN152) strain is nutritionally deficient and unable to synthesize three essential amino acids: leucine, histidine, and arginine. The other strains were engineered with selection markers based on synthetic genes for these amino acids through homologous recombination. All four strains exhibit normal growth on YPD medium. In SC-Leu medium, which contains histidine and arginine but lacks leucine, the WT strain is unable to grow. In SC-Leu-His medium, which lacks both leucine and histidine, neither the WT strain nor the *num11*Δ∷*LEU2* strain can grow. In SC-Leu-His -Arg medium, which is supplemented exclusively with leucine, histidine, and arginine, the *num11*Δ/*NUM11*N strain exhibits normal growth.
