# Supplementary material for: Mitochondrial anchor protein Num11 is key to pathogenicity of Candida albicans by affecting mitochondrial function and cell wall masking
Source: Virulence. 2025 Jun 18;16(1):2519149. doi: 10.1080/21505594.2025.2519149 (PMC12184122; doi:10.1080/21505594.2025.2519149)
Supplement: S1 Fig.docx [file KVIR_A_2519149_SM4320.docx]

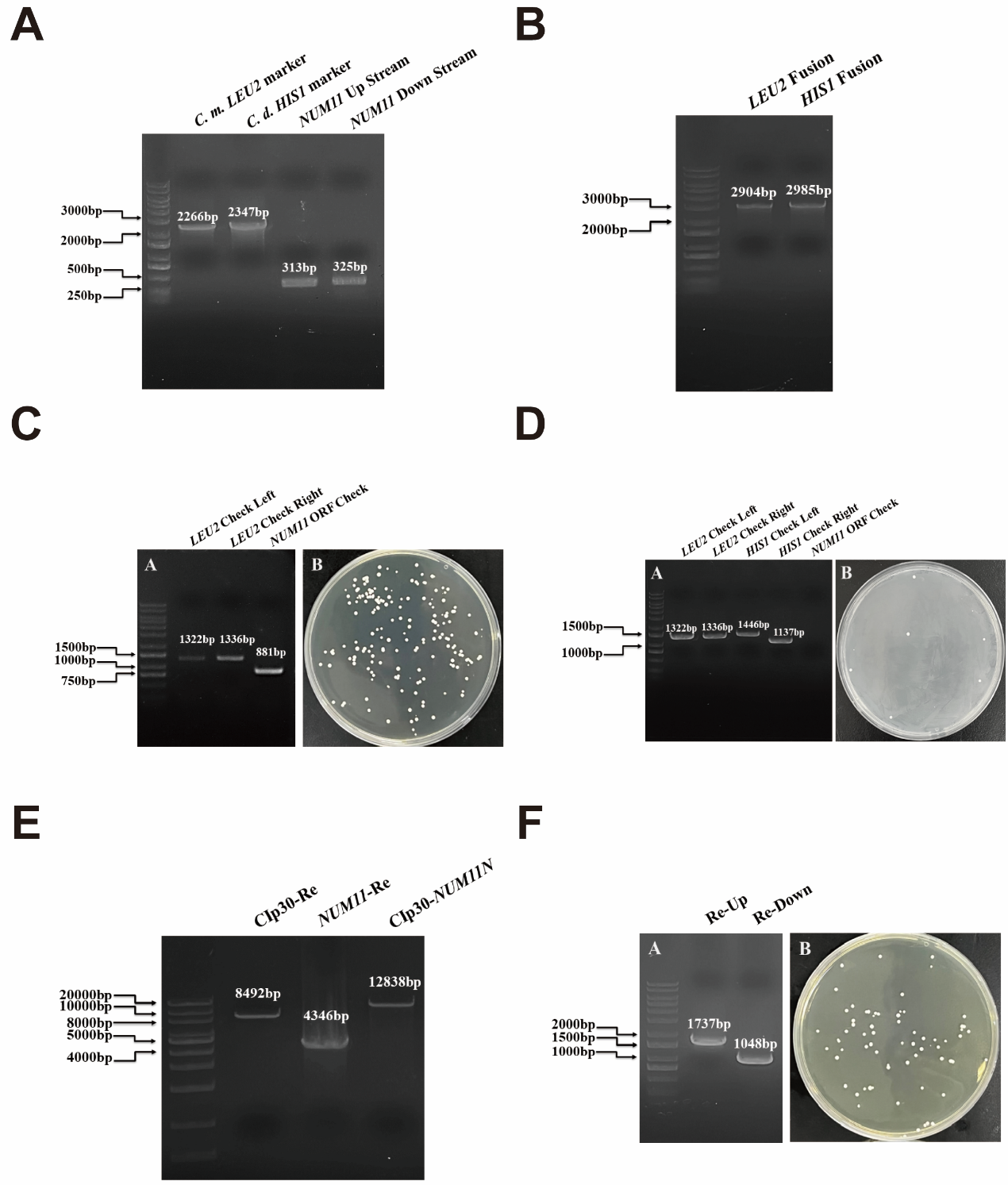


**Fig S1. Identification of *NUM11* gene deletion in *C. albicans*** (A)The upstream and downstream fragments of *NUM11*, along with the *LEU2* and *HIS1* selection marker fragments. (B) The fused upstream and downstream product fragments of *NUM11* and the selection markers. (C) Identification of the *num11*Δ∷*LEU2* strain using suite PCR. (D) Identification of the *num11*Δ/Δ strain using a PCR kit. (E) The CIp30-Num11 promoter fragment and *NUM11* ORF. Recombination of CIp30-Num11 promoter fragment with *NUM11* ORF through *Stu I* single-enzyme cleavage, utilizing their homology. (F) Identification of the revertant strain *num11*Δ/*NUM11*N*.*
