# Supplementary material for: Mitochondrial anchor protein Num11 is key to pathogenicity of Candida albicans by affecting mitochondrial function and cell wall masking
Source: Virulence. 2025 Jun 18;16(1):2519149. doi: 10.1080/21505594.2025.2519149 (PMC12184122; doi:10.1080/21505594.2025.2519149)
Supplement: Table S4.docx [file KVIR_A_2519149_SM4319.docx]

**Table S4. The primers used in qPCR.**

| Gene | Forward primer (5’ to 3’) | | Reverse primer (5’ to 3’) |
| --- | --- | --- | --- |
| *β-actin1*-*candida albicans* | | TGCTGAACGTATGCAAAAGG | TGAACAATGGATGGACCAGA |
| *ALS1* | TTGGGTTGGTCCTTAGATGG | | ATGATTTCAAAGCGTCGTTC |
| *ALS3* | CCTATACCACTGCTACTACCGTTA | | GTATGGTTGGTGTAATGAGGACG |
| *CSH1* | AGTCAAAGACGACGCAGAAG | | TTCAGCTGCGGCTAAGATATG |
| *CHS2* | CCCCACTGAACGTGTCATACCTG | | AACGGACTTCTTGTTGTGGAGGAG |
| *PHR1* | GTCCACCCCACCAGTTGAAGTTG | | GGCATCTTGCTGATAGGCGATACC |
| *BGL2* | AGCTGCTGAAGCTGAAGGATTCC | | ATGGTGGAGACGGAAATCTTTGGC |
| *XOG1* | GTCGCTGGTGAATGGTCTGCTG | | CCCTCATAACGTGCTCCTCTGTTG |
| *OCH1* | GATTGGGCCGATTGGTACGCTAG | | ATTCACGCAACATTGGATGACCTC |
| *CHS3* | AGATTCTATTGCCACCACCGATTAC | | TCAAGTCCGACATCATATCCAACAC |
| *GSC1* | TTGCGTGCTTACCCTGATTTGC | | TACTCTTGGTTCCTCGTCCTCATTC |
| *ALG5* | TTGGTGGTTACTCGATTGGCATATC | | TTCTTGTTAATTCGTCCACATTCATCC |
| *CHS8* | CTCGTCGTGGTCGTAGAAATGGG | | ACCATCGCCATCTTCTTCATCTCC |
| CEK1 | GTTGTTGTTGTTGTTGTGCTTGAGC | | GACGAGGGGAAGAAGAAGTAGTAGC |
| *CDC42* | ATGTGTTGTTGTCGGTGATGGTG | | AATCCCAAGGTAAATGGTTCGTCTC |
| *CST20* | GCGTTTGGCGGTGAGAATAATGC | | GTGGAGGCGGCGGAGGTG |
| *STE11* | AGTATTCTGGATGGCACCTGAAG | | CAAATCTGGGAATGGATGTCTACC |
| *CPH1* | TGCTGCCACTGCTCCAATGTATG | | TTGTTGGTGAGGTGGAATCATGCC |
| *HST7* | CAAGCGGTAGCCTAAGGAGTTCTG | | TAGGTGGCGGGCGTTGTCTC |
| *DFI1* | CGAGTCTGCCAACTGCCTCTAAC | | AACCACCAACCCCAACAACTACAC |
| *β-actin1*-*mouse* | CGTAAAGACCTCTATGCCAACA | | AGCCACCAATCCACACAGAG |
| *IL-6* -mouse | GAAACCGCTATGAAGTTCCTCTCTG | | GTATCCTCTGTGAAGTCTCCTCTCC |
| *TNF-α* -mouse | AAGACACCATGAGCACAGAAAGC | | GCCACAAGCAGGAATGAGAAGAG |
| *IL-6-human* | TTCGGTCCAGTTGCCTTCTCC | | TCTGAAGAGGTGAGTGGCTGTC |
| *TNF-α-human* | TCAGCAAGGACAGCAGAGGAC | | GGTGGAGCCGTGGGTCAG |

All primers were designed by SnapGene Viewer software.
