# Supplementary material for: Mitochondrial anchor protein Num11 is key to pathogenicity of Candida albicans by affecting mitochondrial function and cell wall masking
Source: Virulence. 2025 Jun 18;16(1):2519149. doi: 10.1080/21505594.2025.2519149 (PMC12184122; doi:10.1080/21505594.2025.2519149)
Supplement: Table S6.docx [file KVIR_A_2519149_SM4318.docx]

**Table S6 The MIC of Flu, CAS, AmB, H₂O₂, and DMSO against various strains.**

| Strains | Flu(µg/mL) | | CAS(µg/mL) | AmB(µg/mL) | H_2_O_2_(mM) | DMSO |
| --- | --- | --- | --- | --- | --- | --- |
| WT | | 2 | 0.25 | 0.1 | 6 | 12% |
| *num11*Δ/Δ | | 1 | 0.125 | 0.05 | 6 | 6% |
| *mdm36*Δ/Δ | | 2 | 0.25 | 0.1 | 6 | 6% |
| *dyn1*Δ/Δ | | 0.5 | 0.125 | 0.025 | 6 | 6% |

Minimum inhibitory concentrations (MICs) of Fluconazole (Flu), Caspofungin (CAS), Amphotericin B (AmB), hydrogen peroxide (H₂O₂), and dimethyl sulfoxide (DMSO) against wild-type (WT) and gene deletion strains (*num11*Δ/Δ, *mdm36*Δ/Δ, *dyn1*Δ/Δ). MIC values are expressed in µg/mL for Flu, CAS, and AmB, in mM for H₂O₂, and as percentage (v/v) for DMSO.
