# Supplementary material for: Mitochondrial anchor protein Num11 is key to pathogenicity of Candida albicans by affecting mitochondrial function and cell wall masking
Source: Virulence. 2025 Jun 18;16(1):2519149. doi: 10.1080/21505594.2025.2519149 (PMC12184122; doi:10.1080/21505594.2025.2519149)
Supplement: Table S3.docx [file KVIR_A_2519149_SM4317.docx]

**Table S3. Plasmids used in this study**

| Plasmid | Genotype | Reference |
| --- | --- | --- |
| pSN40 | *C.m.* with *LEU2* screening marker, Kana^r^ | Noble^1^ |
| pSN52 | *C.d.* with *HIS1* screening marker, Kana^r^ |  |
| CIp30 | the integrating vector for *C. albicans*, *Ura3*, Amp^r^ | Dennison^2^ |
| CIp30-*NUM11N* | Part of the length *NUM11N* gene in CIp30 | This study |

1. Noble SM, Johnson AD. Strains and strategies for large-scale gene deletion studies of the diploid human fungal pathogen *Candida albicans*. *Eukaryot Cell.* 2005;4(2):298-309.

2. Dennison PM, Ramsdale M, Manson CL, Brown AJ. Gene disruption in *Candida albicans* using a synthetic, codon-optimised Cre-loxP system. *Fungal Genet Biol.* 2005;42(9):737-748.
