# Supplementary material for: Mitochondrial anchor protein Num11 is key to pathogenicity of Candida albicans by affecting mitochondrial function and cell wall masking
Source: Virulence. 2025 Jun 18;16(1):2519149. doi: 10.1080/21505594.2025.2519149 (PMC12184122; doi:10.1080/21505594.2025.2519149)
Supplement: S4 Fig.docx [file KVIR_A_2519149_SM4316.docx]

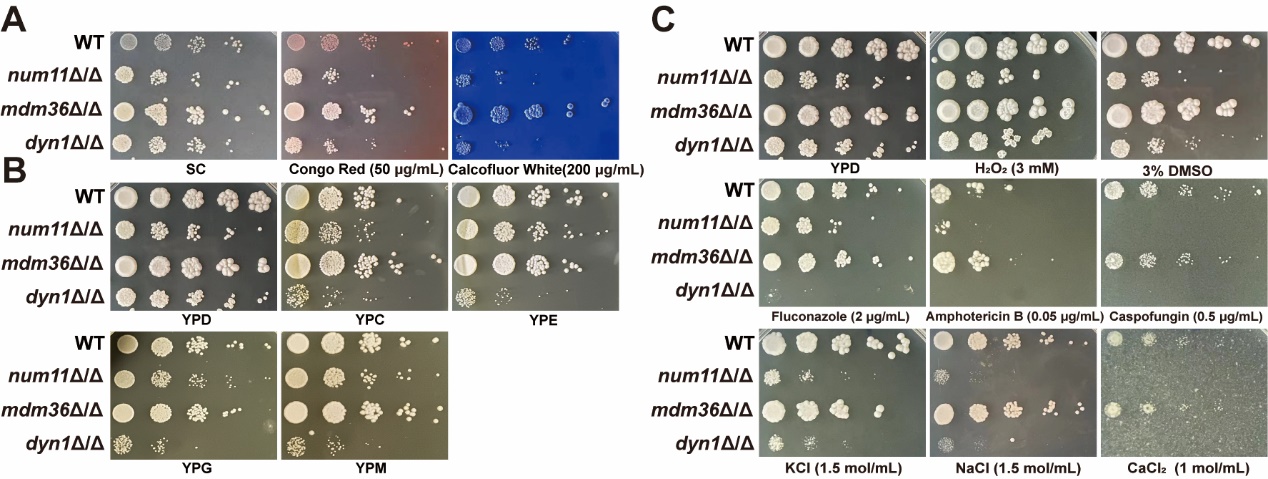


**Fig S4. Carbon Source Utilization and Sensitivity Assays for Various Strains.** (A) Sensitivity of strains to cell wall-perturbing agents. (B) Spot assay to detect carbon source utilization of each strain. (C) Sensitivity of strains to membrane-perturbing agents, drugs, and metal ions. The cells were incubated at 30°C for 2 days. A representative image from three independent experiments with identical results is shown.
